# Detecting local adaptation under weak genetic structure in an endemic damselfly: an integrative eco-evolutionary approach

**DOI:** 10.1186/s12862-025-02462-z

**Published:** 2026-02-02

**Authors:** Pei-Chen Lin, Cheng-Wei Wu, Cheng-Ruei Lee, Jen-Pan Huang, Chung-Ping Lin, Liang-Jong Wang, Yu-Hsun Hsu

**Affiliations:** 1 Department of Life Sciences, National Cheng Kung University, No. 1, University Road, Tainan, 70101 Taiwan; 2 Department of Bioenvironmental Systems Engineering, National Taiwan University, Taipei, 106319 Taiwan; 3 Institute of Ecology and Evolutionary Biology, National Taiwan University, Taipei, 106319 Taiwan; 4 Institute of Plant Biology, National Taiwan University, Taipei, 106319 Taiwan; 5 Biodiversity Research Center, Academia Sinica, Taipei, 11529 Taiwan; 6 Department of Life Science, National Taiwan Normal University, Taipei, 11677 Taiwan; 7 Division of Forest Protection, Taiwan Forestry Research Institute, Taipei, 10066 Taiwan

**Keywords:** Local adaptation, Phenotypic plasticity, Genetic structure, Species distribution modelling, Insect conservation, Genotype-environment association, Climate change resilience, Landscape genomics

## Abstract

**Background:**

Insects comprise one of Earth’s most diverse animal groups, but the adaptive capacity of most species, especially those with weak genetic structure, remains understudied. *Psolodesmus mandarinus* is an endemic damselfly in Taiwan, where its populations show latitudinal variation in wing traits despite limited genetic differentiation in mitochondrial and ribosomal sequences. We hypothesised that weak genome-wide structure may obscure the signals of local adaptation driven by environmental variation. To test this, we integrated genome-wide SNPs, phenotypic measurements, environmental associations, and species distribution models.

**Results:**

Although genome-wide population structure was generally weak, pairwise *F*_ST_ values exceeded 0.35 between southeastern and northeastern populations, and genetic-environment association analyses identified outlier loci and individuals associated with environmental variables. Wing traits, particularly wing colours, exhibited a latitudinal divergence and exceeded expectations from neutral structure (*P*_ST_ >*F*_ST_), indicating selection. Species distribution models showed ecological differentiation and predicted range expansion for clear-winged individuals but range contraction for dark-winged individuals under future climate scenarios.

**Conclusion:**

Our findings demonstrate that phenotypic divergence can arise and persist under weak genetic structure, highlighting the evolutionary potential for local adaptation in structured environments even in species with high dispersal potential. An integrative framework provides essential insights for predicting biodiversity responses to environmental change and guiding climate-resilient conservation strategies.

**Supplementary information:**

The online version contains supplementary material available at 10.1186/s12862-025-02462-z.

## Introduction

Insects are among the most diverse and ecologically important groups of animals on Earth [1], providing crucial services, such as pollination, nutrient cycling, and biological control [2, 3]. However, there are global declines in insect abundance, diversity, and distribution [4, 5] driven largely by climate change, habitat fragmentation, and other anthropogenic pressures [6–9]. Understanding the adaptive process and resilience of insects responding to environmental changes is critical for designing effective conservation strategies.

Despite the scale of these declines, insect conservation remains challenging, with research disproportionately focused on a few charismatic taxa [10], such as butterflies and beetles [11–17]. Increasing attention has recently been given to other groups, including Odonata [18]. Meanwhile, advances in genomic techniques allow us to detect cryptic population structures and evolutionary resilience in understudied insect taxa [18–22], which is especially valuable for understanding how insects respond to environmental stressors. Among the various threats to insects, climate change is particularly pervasive [18, 23]. Altered temperature and precipitation patterns influence habitats, phenology, food resources, and selection regimes [24–27]. These changes may drive adaptive trait shifts [28], but where adaptation is insufficient, populations risk isolation or local extinction [29]. Investigating how phenotypic traits relate to environmental gradients can thus provide insight into local adaptation and vulnerability under future scenarios.

Local adaptation occurs when populations evolve traits that increase fitness in their specific environments [30, 31]. Although it can enhance resilience to environmental changes [32, 33], the outcome depends on the complex interactions among selection, gene flow, genetic drift, and landscape heterogeneity [32, 34, 35]. Understanding when and how local adaptation arises remains an ongoing challenge in conservation biology [34–37].

**Fig. 1:**
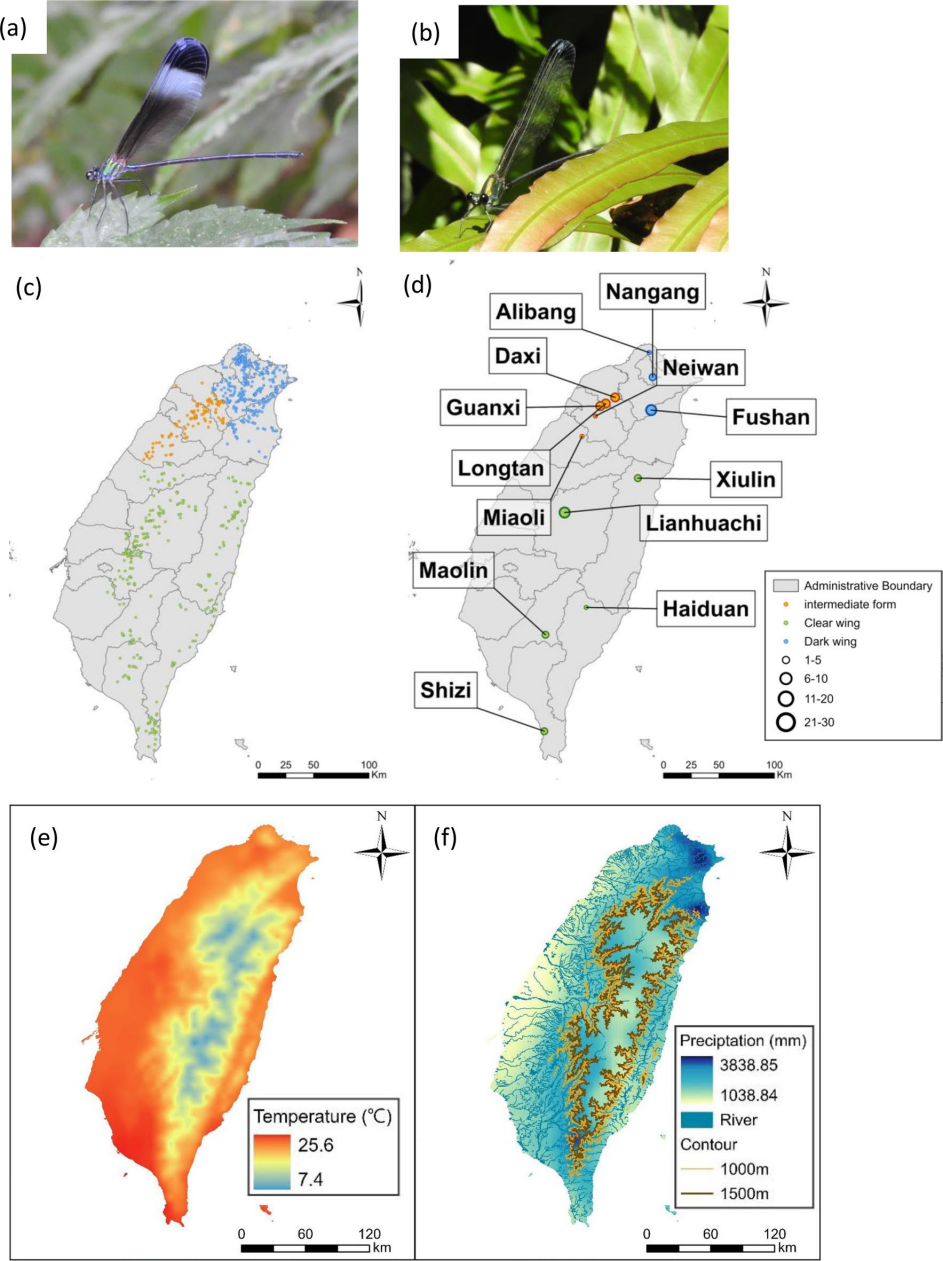
*Psolodesmus mandarinus*: (**a**) a dark-winged male, (**b**) a clear-winged male. (**c**) presence records used for species distribution modelling (one point per location), and (**d**) collection sites for genomic and phenotypic analyses, with circle size indicating sample size (detailed counts in Table S2). Photos taken by Chun-Yu Kuo. Spatial distribution of climate variables in Taiwan (2013–2023 averages gridded monthly observational data from the Taiwan Climate Change Projection and Information platform, TCCIP): (**e**) mean annual temperature (°c), with warmer lowlands in the west and cooler high-elevation regions in the central and eastern mountains; and (**f**) mean annual precipitation (mm), highlighting higher rainfall in the east and central mountain ranges compared to the drier western plains, shown with major rivers from the Water Resources Agency, Ministry of Economic Affairs (MOEA), and elevation contours (1000 m and 1500 m) derived from a 100 m DEM provided by the ministry of the Interior (MOI) using the contour tool in ArcGIS pro. All maps were generated in ArcGIS pro (version 3.3, Esri, Redlands, CA, USA)

*Psolodesmus mandarinus* is a damselfly species endemic to the main island of Taiwan, and one of only two described species in the genus *Psolodesmus*, which is otherwise restricted to the Japanese Ryukyu Archipelago [38, 39]. Males defend territories along stream margins where females oviposit [40], making stream-associated microhabitats essential for reproduction. This species exhibits a pronounced latitudinal gradient in wing colouration: northern populations displaying black, white, and brown colours from apical to basal areas on their wings (dark-winged individuals; Fig. 1a), while central and southern populations have predominantly transparent wings with a thin apical blackish margin (clear-winged individuals; Fig. 1b) [39, 41, 42]. Our previous behavioural studies further revealed that males of the two wing forms differ in territorial displays, suggesting that wing colours might play a role in intraspecific signalling [40]. Despite these marked phenotypic differences, earlier mitochondrial and ribosomal DNA studies, both primarily designed for species identification, found little genetic differentiation between the forms [38, 42, 43], and no corresponding divergence in their parasite communities [44]. These findings raise questions about the evolutionary and ecological basis of the observed phenotypic variation.

Although few studies have directly tested how climate shapes wing traits in damselflies, pigmentation and morphology are known to influence fitness in Odonata [45–48]. Darker wings may be advantageous in cooler environments, as they absorb solar radiation more efficiently in accordance with the thermal melanism hypothesis [45, 49, 50], although some studies reported non-supporting evidence [51, 52]. In addition to potential thermal effects, wing pigmentation can play roles in mate signaling and sexual selection [48], with darker males often achieving higher mating success [46, 47]. These findings suggest that wing colouration may mediate responses to environmental and social variation, but its association with climatic gradients remains poorly understood. Therefore, the latitudinal forms of *P. mandarinus* provide a valuable opportunity to examine whether environmentally associated phenotypic divergence can arise under weak genetic differentiation.

With recent advances in population genomics and genotype-environment association (GEA) analyses [53–55]. *P. mandarinus* is well-suited to investigate the genomic and environmental basis of adaptation in an understudied, endemic insect. In this study, we integrate genome-wide single-nucleotide polymorphism (SNP) data, phenotypic trait measurements, and species distribution models to evaluate population structure, detect signals of local adaptation, and investigate the effects of climate change by evaluating potential range shifts under future climate scenarios. We hypothesis that weak genome-wide population structure may obscure local adaptation driven by environmental variations. To test this, we predict: (1) a weak genome-wide population structure with outlier loci associated with environmental variations; (2) greater divergence in wing traits than expected under neutral genetic structure, with association to environmental variables; and (3) different ecological niches between wing forms, leading to contrasting projected range shifts under climate change.

## Methods

### Data collection

#### Field data collection

We surveyed 153 stream and creek locations ( < 3 m wide) across Taiwan during the adult flight season (March–November) to locate *P. mandarinus* populations. Wing characteristics were recorded for each observed individual. We collected 5–20 adults per population using nets, but limited our collection to 2–3 individuals (~10% of the observed individuals) in smaller populations ( < 20 individuals) to minimise impact. Captured individuals were photographed alive for wing measurements and preserved in 95% ethanol for DNA extraction.

For form-specific analyses, individuals were classified into three wing types following previous taxonomic descriptions [39]: (1) dark-winged, characterised by a whitish semiopaque transverse fascia (“white band”) and a brownish sub-basal area; (2) clear-winged, lacking both the white band and the brownish sub-basal area; and (3) intermediate form, including individuals without a white band but with a brownish sub-basal area, as well as those from transition zones (Daxi, Longtan, Guanxi, Neiwan and Miaoli, also referred to as intermediate populations for subsequent analyses; Fig. 1) along the latitudinal gradient of wing morphology [42].

#### Data collection for species distribution modelling

We recorded and georeferenced 1,159 adults of *P. mandarinus* in our field surveys across Taiwan’s main island. To improve spatial coverage, we supplemented our dataset with occurrence records from two citizen science platforms: (1) iNaturalist (https://www.inaturalist.org), where we retrieved 1,235 presence records (accessed on 20^th^ Feb 2024 [56]), and (2) Nature Campus (http://nc.biodiv.tw/bbs/), from which we extracted 1,508 records after screening 17,136 Odonata survey entries (accessed on 20^th^ Feb 2024). Each individual was categorised into dark-winged, clear-winged, or intermediate form based on observed wing morphology.

iNaturalist records included precise GPS coordinates, while Nature Campus data provided descriptive locations, which we georeferenced using Google Earth. To enhance the accuracy of the niche model, we excluded records with unclear locations, insufficient details, or unverifiable coordinates. We retained only one presence record per 100-meter radius to minimise the possibility of repeated sampling, as most observations were from unmarked individuals. The final dataset comprised 664, 426, and 177 presence records for the dark-winged, clear-winged, and intermediate forms, respectively (Fig. 1c).

The environmental data comprised the full set of 19 bioclimatic variables related to temperature and precipitation (listed in Table S1), were obtained from CHELSA (https://chelsa-climate.org/) at a 2.5-minute resolution. To assess potential distribution shifts under future climate change, we incorporated three Shared Socioeconomic Pathways (SSP126, SSP370, and SSP585) across three time periods (2011–2040, 2041–2070, and 2071–2100) based on projections from the Beijing Climate Center Climate System Model (BCC-CSM2-MR) under CMIP6 [57]. These scenarios represent distinct socio-economic trajectories: SSP126 follows a sustainability pathway with net-zero CO₂ emissions by 2075, SSP370 reflects a fragmented world with increasing emissions, and SSP585 projects fossil-fuel-driven growth with the highest emissions by 2100. By incorporating multiple timeframes and contrasting climate scenarios, this analysis comprehensively assesses potential distribution shifts and their implications for different wing forms.

### From RADseq to SNPs filtering

Genomic DNA was extracted from thoracic muscle tissue using the DNeasy Blood & Tissue Kit (Qiagen) from 141 *P. mandarinus* individuals collected from 13 populations across Taiwan during our field survey to cover multiple populations of different wing forms (Fig. 1d, Table S2). Restriction-site Associated DNA Sequencing (RADseq) library preparation followed the multiplexed shotgun genotyping protocol [58]: DNA samples (50 ng each) were digested with *MseI*, barcoded, and size-selected (300–450 bp) using Pippin Prep (Sage Science, USA). Libraries were amplified via 14 PCR cycles and sequenced on an Illumina NovaSeq 6000 platform (150 bp paired-end reads) at Welgene Biotech Co. (Taipei, Taiwan). The de-multiplexed RADseq data were filtered and assembled *de novo* using ipyrad v0.9.61 [59] due to the absence of a reference genome. Sequence reads were filtered to remove 5-nt barcodes, *MseI* cut sites (TAA), low-quality reads (Phred score Q < 20), and clustering loci with a similarity threshold of 0.85. Loci with an average coverage of fewer than six reads per individual were excluded. After filtering, samples with fewer than 100,000 retained reads were excluded from further analysis to minimise bias due to inconsistent sequencing depth.

For downstream population genomic analyses, we applied a locus completeness threshold 70% (i.e., each SNP genotyped in ≥ 70% of individuals), and retained the complete SNP dataset without additional filtering, such as Hardy–Weinberg equilibrium (HWE), linkage disequilibrium (LD), or minor allele frequency (MAF). While such filters are commonly applied to minimise genotyping errors, they can also disproportionately remove loci that capture weak signals of population differentiation, particularly under conditions of high gene flow and limited genetic structure [60]. To ensure that potentially informative loci were retained, we based our main analyses on unfiltered datasets. To evaluate robustness, we additionally generated more conservative datasets by applying HWE filtering, LD pruning (r^2^ > 0.5), and MAF < 0.01, using PLINK v1.9 [61], as detailed in Supporting Information 2. Finally, to test sensitivity to the completeness threshold itself, we repeated population structure analyses with a relaxed 50% threshold, presented in Supporting Information 3.

### Population structure analyses

Based on the full SNP dataset, we calculated pairwise *F*_ST_ and examined population structure with STRUCTURE v2.3.4 [62]. An admixture model with correlated allele frequencies was applied, testing values of *K* from 2 to 13, corresponding to the number of sampled populations. Each run consisted of 500,000 burn-in iterations followed by 500,000 MCMC iterations, with three replicates per *K* value. The optimal *K* was determined using the delta *K* method [63] in Structure Harvester [64]. Results were analysed in CLUMPAK [65] to identify major modes, which were aligned in CLUMPP v1.1.2b [66] and visualised using *Distruct* [67]. To further explore population structure without assumptions of population processes (e.g. LD, HWE) as required in STRUCTURE [68], we first standardised SNP data by imputing missing values with the mean, followed by centring and scaling. The standardised SNP dataset was then used for a principal component analysis (PCA) to explore genetic variation among populations.

To align genetic variation with geographical coordinates, we applied Procrustes rotation of PC1 and PC2 using the package *VEGAN* [69] in R. To assess factors influencing population structure, we evaluated isolation by distance (IBD) [70] and isolation by environment (IBE) [71] with Mantel tests, with genetic distance calculated as multi-locus *F*_ST_/(1-*F*_ST_). Given Taiwan’s north-south orientation and central mountain range, topographic barriers may influence population connectivity. We subdivided populations into eastern and western groups for IBD and IBE analyses to account for potential isolation effects. The western group comprises 10 populations: Alibang, Nangang, Longtan, Daxi, Guanxi, Neiwan, Miaoli, Lianhuachi, Maolin, and Shizi. The eastern group includes six populations: Alibang, Nangang, Fushan, Xiulin, Haiduan, and Shizi. Due to their central locations, three populations (Alibang, Nangang, and Shizi) were included in both. Geographical and environmental distances were calculated as Euclidean distances with packages *REAT* [72] and *VEGAN* in R, with environmental distances derived from the niche models.

Additionally, piecewise structural equation modelling (Piecewise SEM) was employed to disentangle environmental and geographical influences on population structure [73, 74]. This method divides the analysis into smaller, interpretable models and accounts for spatial autocorrelation using random effects [75]. Distances were standardised, and model fit was evaluated using marginal and conditional *R*^2^ values in packages *lme4* and *piecewiseSEM* [73, 76] in R.

### Genotype-environment association (GEA) analyses

Redundancy analysis [RDA; 77]; was conducted using the package *VEGAN* in R to detect multi-locus associations with multivariate environmental variables. We applied RDA to the full SNP dataset for SNP and individual levels to examine genotype-environment relationships. To refine the model, we used forward selection, which sequentially adds explanatory variables based on their significant contribution to the total explained variance, ensuring that only the most relevant variables were retained. Candidate environmental variables were derived from the niche model described earlier. To account for spatial structure, we used Principal Coordinates of Neighbour Matrices (PCNM) analysis [78], which was based on geographic distance matrices calculated from the latitude and longitude coordinates of sampling sites. We retained the first five principal axes from the resulting spatial eigenvectors (PCNM1-5), which captured the most significant spatial variation for later analyses. This approach accounts for spatial structure and is well-suited to detecting weak multi-locus molecular signatures, surpassing the capabilities of single-locus analyses [54]. The analysis of all *P. mandarinus* populations identified three constrained axes that explained over 68% of the variance. Candidate SNPs potentially under local adaptation were selected based on their loadings exceeding three standard deviations from the mean [79, 80]. Individuals were then ranked by their cumulative allele counts at these candidate loci, and the top 10% were identified as candidate individuals likely exhibiting locally adaptive genomic patterns [81].

To evaluate the robustness of the RDA results and explore non-linear environmental interactions, we employed the Gradient Forest model with the full SNP dataset using the package *gradientForest* [82] in R. This model captures complex relationships between environmental predictors and allele frequency shifts across landscapes, enabling quantification of the cumulative importance of each environmental variable. The insights gained through Gradient Forest provided an additional perspective on how environmental gradients influence genetic structure, complementing the findings from the RDA.

### Phenotype-environment association analyses

Phenotypic traits, including the wing size (WS) and the ratio of apical blackish area of the whole wing (ABA%), both reported to show a latitudinal gradient in *P. mandarinus* [42], were measured from 137 individuals collected across 13 populations during our field survey for genomic analyses (Fig. 1d, Table S2). Most of the specimens included for phenotypic measurement are the same specimens used for genomic analyses, except for three specimens used in genomic analyses but not phenotypic analyses, and one specimen used in phenotypic analyses but not genomic analyses. We calculated Pearson’s correlation coefficients to ensure these two traits are not highly correlated (*r* < 0.6).

To evaluate whether phenotypic variation was driven by selection or genetic drift, a comparison between phenotypic and genetic variation (*F*_ST_) was conducted [83, 84]. Here, the phenotypic variation was represented by *P*_ST_, which served as a proxy for *Q*_ST_ due to the lack of pedigree data [85]. Following this equation

1 $${P_{ST}} = {{c{V_B}} \over {2{h^2}{V_W} + c{V_B}}}$$

where *V*_*B*_ is the phenotypic variance between populations, *V*_*W*_ is the phenotypic variance within populations, *h*^*2*^ is the narrow-sense heritability (the proportion of phenotypic variance due to additive genetic effects), and *c* is a scaling factor for the among-population component. We calculated and evaluated the sensitivity of *P*_ST_ for each phenotypic trait across a range of plausible genetic variance scenarios (*c*/*h*^2^) by varying the proportion of additive genetic variance (*c*) and the narrow-sense heritability (*h*^2^) independently from 0.1 to 1 in increments of 0.1. Under the null assumption that *c* = *h*^*2*^, *P*_ST_ can be directly compared to *F*_ST_ to evaluate whether phenotypic differentiation exceeds neutral genetic expectations. The intersection of the *P*_ST_ and *F*_ST_ confidence intervals (CI) was used to determine the critical c/*h*^*2*^ value, at which the lower 95% CI of *P*_ST_ equals the upper 95% CI of *F*_ST_.

Random Forests [RF; 86] were used to explore associations between traits and environmental variables, enabling the detection of non-linear responses and interactions [87, 88]. To allow a direct comparison with GEA results, the two phenotypic traits were combined into a single composite variable using PCA. The first principal component (PC1), explaining 78% of the variation, was used in subsequent analyses. Environmental variables considered in this analysis mirrored those used in the GEA analyses, ensuring consistency across methodologies.

### Species distribution modelling and niche similarity tests

To predict the potential distribution of *P. mandarinus* and that for each of its wing forms, we employed the Maximum Entropy Methodology using MaxEnt 3.3.33 [89] with presence-only data. Three datasets were analysed: (1) dark-winged individuals (i.e., individuals with dark-winged form), (2) clear-winged individuals (i.e., individuals with clear-winged form), and (3) all individuals, including dark-winged, clear-winged, and intermediate forms. To reduce autocorrelation among environmental variables, pairwise Spearman correlations were calculated using the package *ENMTools* [90] in R. For correlated variables (|r| > 0.8), we retained those with higher model contributions [91]. Variables contributing < 1% to the model were also excluded to prevent overfitting [92] to establish the final variable combination of each model (Table S1). We then assessed the model performance of each model by calculating the Area Under the Receiver Operating Characteristic Curve (AUC) [93], with higher AUC values indicating better predictive performance [94].

A niche similarity test was conducted using Schoener’s *D* [95] and Hellinger’s *I* [96] to evaluate niche differences between dark- and clear-winged individuals. We used ENMtools to calculate the two indices based on 10,000 background points. To assess statistical significance, a permutation-based null distribution was generated by randomly reassigning phenotype labels (dark- or clear-winged) among individuals and recalculating *D* and *I* values across 100 permutations [90]. A significant difference was inferred if the observed *D* and *I* values were lower than the 95% CI of the null distribution, suggesting divergent environmental niches between the two phenotypes.

## Results

### Population structure analysis

Illumina sequencing yielded approximately 362 million sequence reads across 137 individuals. After demultiplexing with the *de novo* method, ~7.3 million consensus reads, and 14,763 loci were assembled. Applying a 70% completeness threshold substantially reduced the dataset to 656 loci for downstream analyses.

**Fig. 2:**
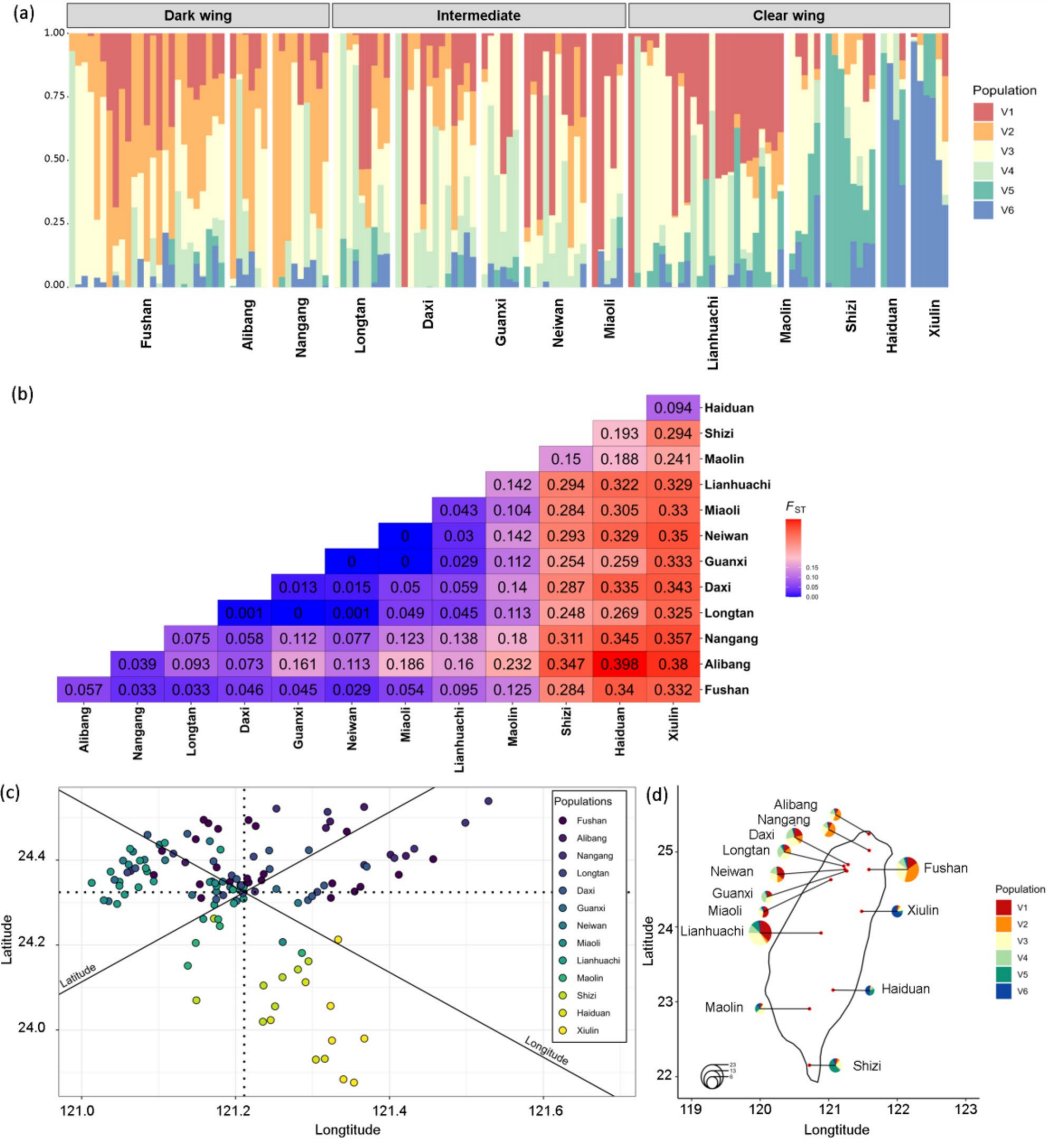
The genome-wide population structure of *Psolodesmus mandarinus*. (**a**) STRUCTURE results for the full SNP dataset (*K* = 6), showing weak overall structure and extensive admixture despite a large value of optimal *K*. (**b**) Estimated pairwise *F*_ST_ values among populations. (**c**) Procrustes PCA ordination aligning genetic variation with geography, coloured by sampling locations from north (purple) to south (yellow). (**d**) Geographic map linking STRCUTURE results to sampling locations, illustrating the association between population structure and geography. Across methods, southeastern populations (Xiulin, Haiduan, Shizi) consistently showed stronger differentiation from the other populations

STRUCTURE analysis revealed weak genome-wide structure and extensive admixture, with an optimal *K* of 6 for the full dataset (Fig. 2a). Genetic variation followed a gradual latitudinal cline rather than forming distinct population clusters, with southeastern populations (Xiulin, Haiduan, and Shizi) showing stronger differentiation. Pairwise *F*_*ST*_ values indicated generally moderate to low genetic differentiation among populations (Fig. 2b). The strongest differentiation was observed between the northernmost population (Alibang) and the eastern populations (Haiduan and Xiulin), with *F*_ST_ ≥ 0.38. The southeastern population (Shizi) also showed relatively high differentiation from northern and northwestern populations, with *F*_ST_ values mostly greater than 0.25.

Conventional PCA supported these results, with the first two principal components explaining less than 20% of the variation and showing no clear clustering of populations (Figure S8a in Supporting Information 2). Procrustes PCA aligned genetic variation with geography and confirmed the latitudinal gradient without assuming population-level processes such as LD or HWE (Fig. 2c). Finally, mapping STRUCTURE results onto geography further highlighted the association between genetic variation and sampling locations (Fig. 2d).

**Fig. 3:**
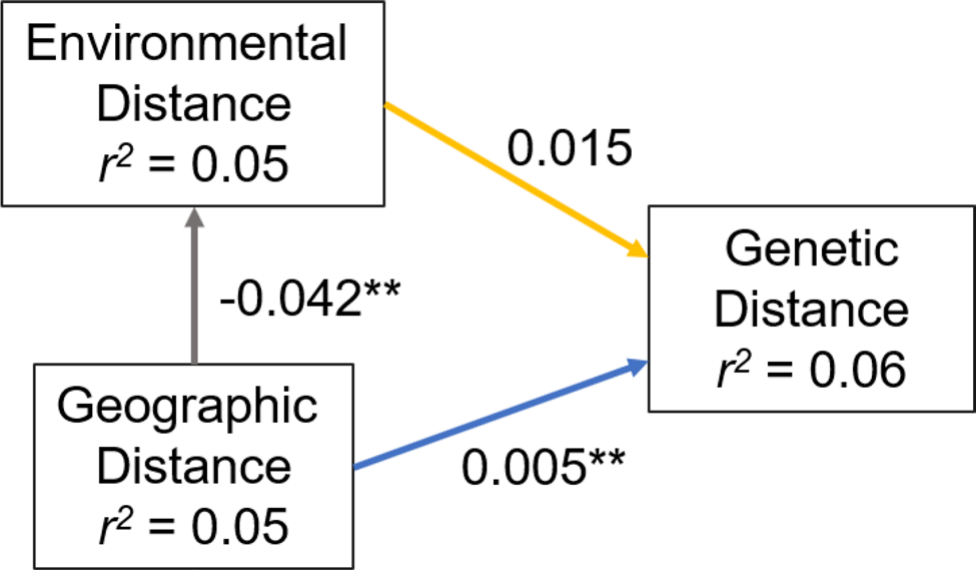
Piecewise structural equation modelling (Piecewise SEM) results illustrate geographic, environmental and genetic distance relationships. Arrows indicate modelled paths, with values representing standardised path coefficients (i.e., standardised regression coefficients), with statistical significance indicated as *p* < 0.05 (*), *p* < 0.01 (**), *p* < 0.001 (***). The *r*^2^ values indicate the proportion of variance explained in each response variable

Mantel tests across all populations showed weak correlations, with neither IBD (*r* = 0.21, *p* > 0.05) nor IBE (*r* = 0.02, *p* > 0.05) being significant. Regional analyses revealed contrasting patterns: in eastern populations, IBD correlations were relatively strong (*r* = 0.70) but not significant, whereas IBE was significant (*r* = 0.75–0.79, *p* < 0.05). In western populations, neither IBD nor IBE was detected. Piecewise SEM further indicated that genetic distance was primarily explained by geographic distance, with no independent effect of environmental distance (Fig. 3). Although geographic and environmental distances were not correlated across all populations (*r* = −0.22, *p* > 0.05), they were strongly correlated within regions (East populations: *r* = −0.96, *p* < 0.001; West populations: *r* = −0.24, *p* < 0.001).

### GEA analysis

**Fig. 4:**
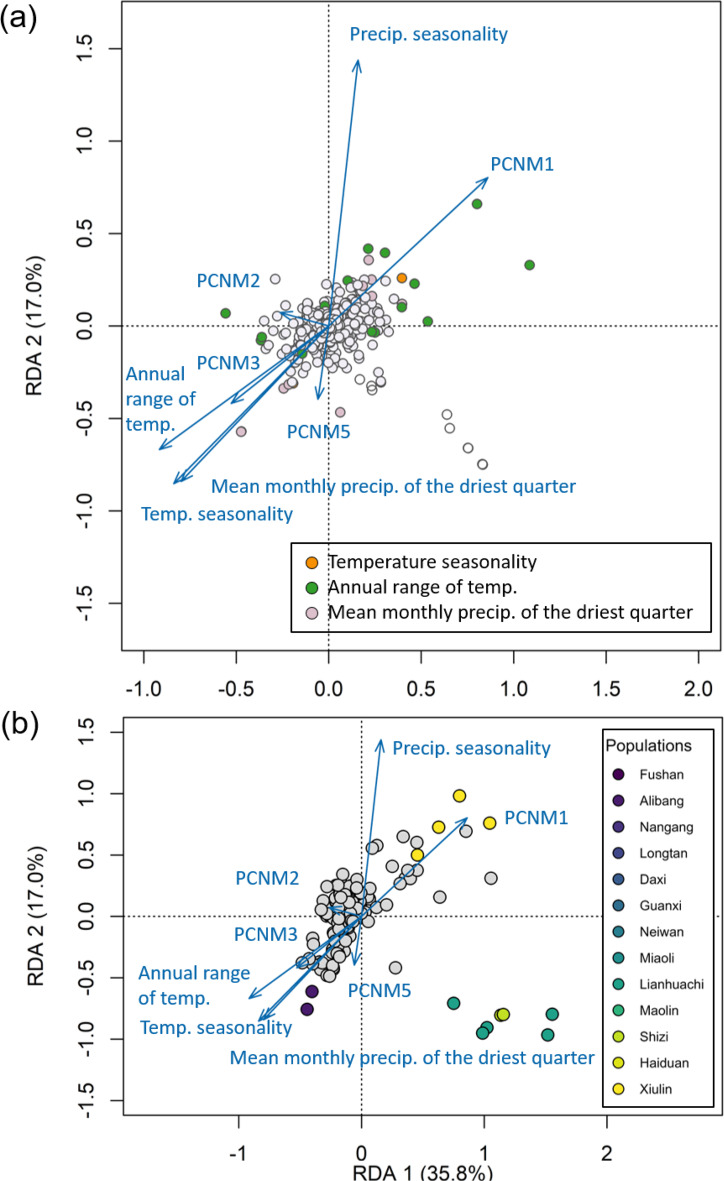
Redundancy analysis (RDA) of *Psolodesmus mandarinus* genetic variation in relation to environmental and spatial predictors. Panel (**a**) presents SNP loadings, and panel (**b**) displays individual scores. Arrows represent the direction and strength of correlations between predictor variables and the RDA axes; longer arrows indicate stronger associations. In (**a**), coloured points denote SNPs most strongly associated with specific predictors, while in (**b**), coloured points represent individuals from different populations most strongly associated with particular environmental or spatial variables. Predictor variables include climatic factors (e.g., temperature seasonality, mean monthly precipitation of the driest quarter) and spatial components derived from PCNM analysis (PCNM1–5)

After the forward selection of variables, the final set of variables in our RDA analysis included climatic factors (temperature seasonality, precipitation seasonality, annual range of temperature and mean monthly precipitation of the driest quarter) and spatial predictors (the first five spatial axes derived from PCNM analysis, PCNM1–5). The first three RDA axes explained 35.8%, 17.0%, and 15.6% of the total genetic variation, respectively (Fig. 4). Across these axes, 19, 12, and 7 outlier loci were identified, respectively (Fig. 4a), with 15 of these outliers also identified through Gradient Forest analysis, suggesting concordant signals of different GEA analyses. These environmental and spatial gradients were further reflected at the individual level (Fig. 4b). Specifically, individuals from the southeastern populations (Xiulin, Haiduan, and Shizi) were clearly separated along PCNM1, which captured major spatial variations and was also associated with precipitation seasonality. In contrast, individuals from the northern to central populations (e.g., Alibang, Nangang, Daxi, Longtan, Neiwan, and Miaoli) were distributed along gradients of temperature seasonality, annual range of temperature, and mean monthly precipitation of the driest quarter (Fig. 4b). The variance partitioning analysis grouped predictors into environmental (i.e., bioclimatic) and geographical (i.e., spatial PCNM1-5) categories to assess their relative contributions to genetic variation. The results showed that environmental factors explained a larger proportion of the independent variance (env. PVE = 4.5%) than geographical factors (geo. PVE = 1.7%) (Table S3). The contribution of all predictors in the RDA model was significantly supported by permutation tests (*n* = 1000) [97].

### Phenotype-environment association analyses

There are significant differences in wing size (WS) and the ratio of apical black area on the whole wing (ABA%) among populations (WS: ANOVA *F* = 3.34, *p* < 0.001; ABA%: ANOVA *F* = 24.61, *p* < 0.001; Figure S1). In addition, *P*_ST_ -*F*_ST_ comparisons showed that both traits showed significantly higher phenotypic differentiation than expected under neutral genetic variation (*F*_ST_ = 0.013, 95% CI = 0.0118 ~ 0.0145), with *P*_ST_ > *F*_ST_ for both WS (*P*_ST_ = 0.705) and ABA% (*P*_ST_ = 0.935); Table 1). The critical value of the *c*/*h*^2^ ratio was lower for ABA% than WS (Table 1).

**Table 1:** *P*_ST_ -*F*_ST_ comparisons of wing size (WS) and the ratio of apical blackish area on the whole wing (ABA%) of *P. mandarinus* among populations

Phenotype	*c*/*h*^2	*P*_ST_	Lower 95% C.I.	Upper 95% C.I.	Critical *c*/*h*^2^	*P*_ST_ - *F*_ST_
WS	1	0.705	0.580	0.803	0.01	*P*_ST_ > *F*_ST_
ABA%	1	0.935	0.899	0.961	0.002	*P*_ST_ > *F*_ST_

**Fig. 5:**
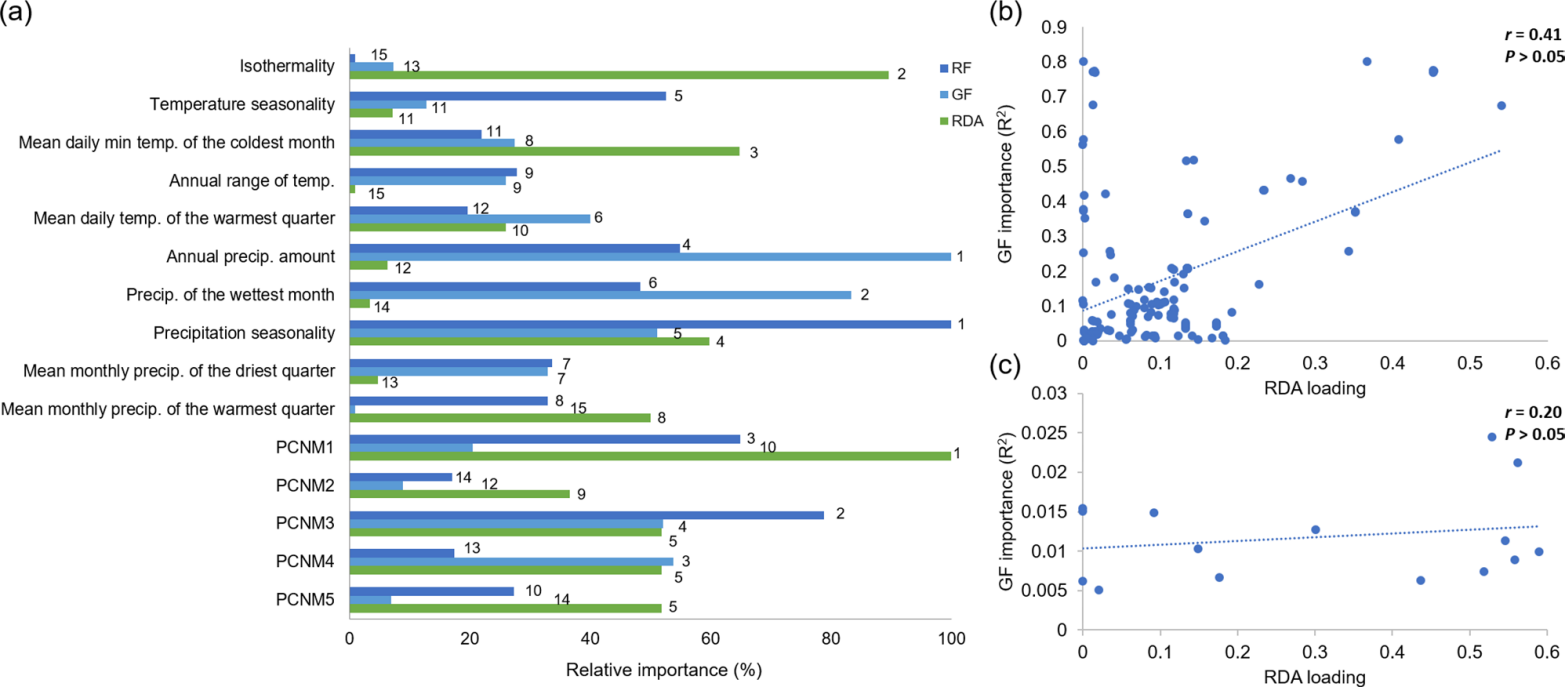
Comparison of environmental predictor importance across methods. (**a**) Relative importance (%) of environmental predictors from redundancy analysis (RDA), gradient forest (GF), and random forest (RF), with numeric ranks indicated beside the bars. (**b**) relationship between RDA loadings and GF importance for SNP loci. (**c**) relationship between RDA loadings and GF importance for environmental predictors. In (**b**) and (**c**), each point represents an SNP or predictor, respectively; dashed lines show regression trends in both

To assess whether similar environmental factors influenced phenotypic and genetic variation, we compared the relative contribution of each variable (scaled from 0 to 1) across RDA, Gradient Forest, and Random Forest (Fig. 5a). Precipitation-related variables (annual precipitation, precipitation seasonality, and precipitation of the wettest month) consistently ranked among the most influential predictors across the three approaches, while spatial predictors (e.g., PCNM1 and PCNM3) also showed strong contributions in RDA and RF. In contrast, some temperature-related factors (e.g., isothermality, minimum temperature of the coldest month) were highly important in RDA but less so in GF and RF. Despite these differences in specific contributions, the overall ranking of predictors was broadly consistent across methods (Friedman test: *χ*^2^ = 0.13, *df* = 2, *p* = 0.94). Nevertheless, no significant correlations were detected between genetic variation and environmental predictors (Fig. 5b, c). Together, these results suggest that both genetic and phenotypic variation in *P. mandarinus* are shaped by partially overlapping environmental gradients, with precipitation and spatial structure emerging as common drivers.

### Robustness of our results

Among the 656 loci available in the full dataset used in the main text, some loci were removed across different filtering approaches, and only 219 loci were retained after applying the Strict filtering strategy (with different combinations of HWE filtering, LD pruning, and MAF). Analyses of LD-pruned and other filtered datasets (Supporting Information 2) resulted in lower estimates of genetic differentiation and reduced the optimal *K* in STRUCTURE analyses. However, the pattern of the results remained consistent with the unfiltered dataset. To further test sensitivity, we repeated population structure analyses under a more relaxed 50% completeness threshold (Supporting Information 3). These also showed consistent results, confirming that our main conclusions are robust to both filtering approaches and completeness threshold.

### Species distribution models

Distribution models show high predictive accuracy [98] with average training AUC values of 0.95 (dark-winged), 0.88 (clear-winged), and 0.90 (all populations). For dark-winged populations, precipitation of the driest quarter (bio17) and temperature seasonality (bio4) were the most influential variables, jointly contributing over 70% to the model (Figure S2a). Clear-winged populations were mainly influenced by the mean temperature of the warmest quarter (bio10), annual precipitation (bio12), and precipitation seasonality (bio15), jointly contributing 81.7% to the model (Fig. S2b). The all-populations model retained the top two variables from each phenotype-specific model, jointly contributing over 84% (Figure S2c). These selected variables for all populations were used in downstream GEA analyses.

**Fig. 6:**
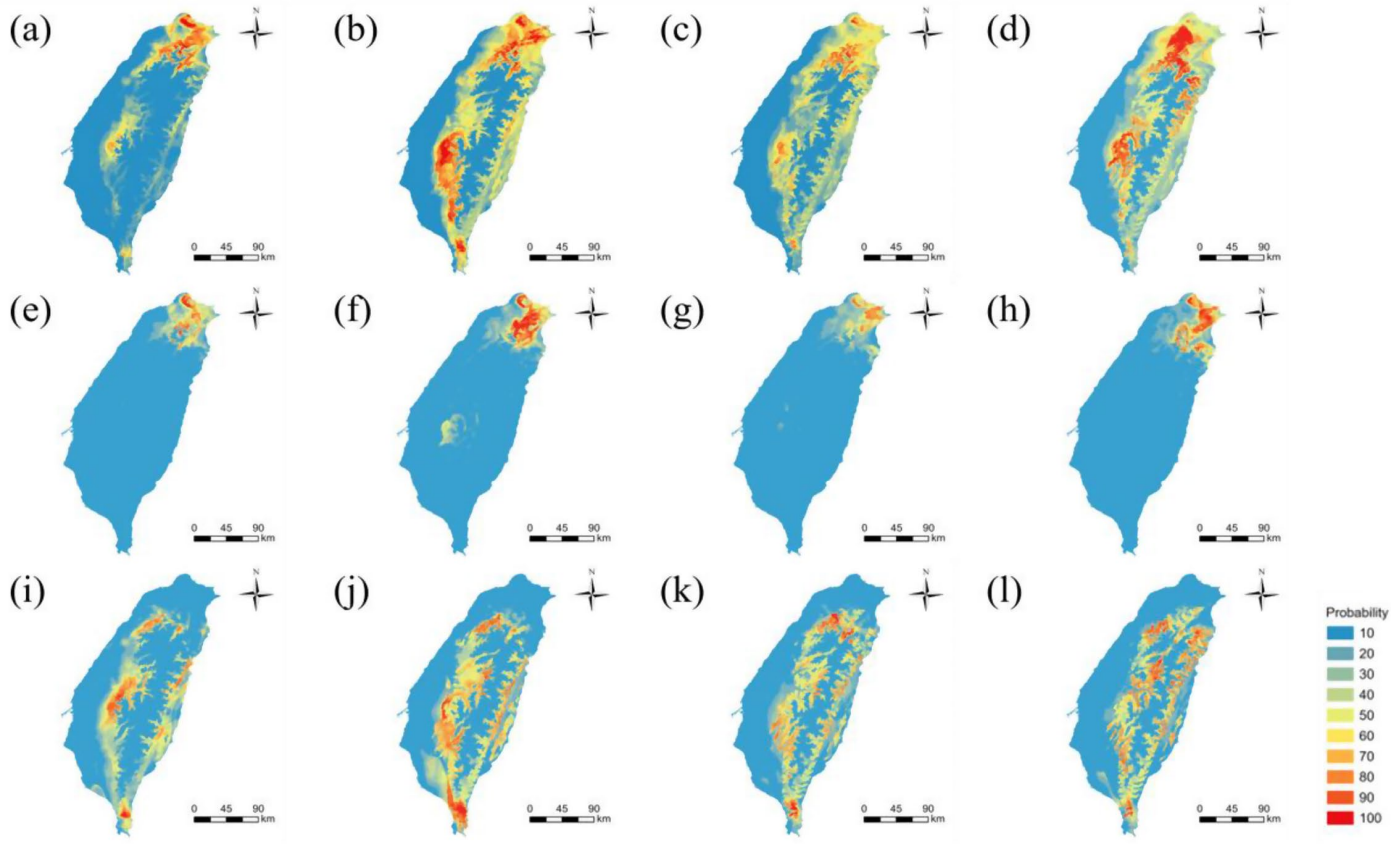
The predicted distributions of *Psolodesmus mandarinus*. Rows correspond to (top) all individuals, (middle) dark-winged individuals, and (bottom) clear-winged individuals. Columns show (**a, e, i**) current distribution, and (**b–d, f–h, j–l**) projected distributions in 2071–2100 under SSP126, SSP370, and SSP585, respectively. Warmer colours indicate higher occurrence probability. Results for intermediate periods (2011–2040 and 2041–2070) are shown in Figures S3–S5

Future projections suggest an overall expansion of *P. mandarinus* by 14–121% across SSP scenarios, with the most pronounced expansions under SSP126 (~121%) and moderate increases under SSP585 (~106%; Table S4). Expansion is concentrated in the central and eastern mountainous regions, reflecting potential upslope and northward shifts (Fig. 6 & S3). However, models for the dark- or clear-winged individuals indicate that this trend is primarily driven by clear-winged individuals (up to 102%; Figures 6 & S4; Table S4), whereas dark-winged individuals exhibit limited northward shifts and even range contraction under SSP370 (−4 to −22%), likely constrained by available northern habitats (Fig. 6 & S5; Table S4). Niche similarity tests further support these differences, as Schoener’s *D* (0.294) and Hellinger’s *I* (0.561) between dark- and clear-winged individuals were significantly lower than their critical values (0.876 and 0.978, respectively), confirming distinct fundamental niches between the two phenotypes.

## Discussion

Understanding the mechanisms of species resilience is essential for evolutionary biology and climate-resilient conservation [55, 99, 100], especially for insects due to their diversity and roles in ecological services [2, 3]. We hypothesised that weak genome-wide population structure in the endemic damselfly *P. mandarinus* may obscure signals of local adaptation driven by environmental variation. By integrating population genomics, GEA, phenotypic-environment associations, and species distribution modelling, we demonstrate that despite weak genome-wide population structure, *P. mandarinus* exhibits regionally structured phenotypic divergence consistent with local adaptation.

### Local adaptation beyond weak population structure

Consistent with our first prediction, we observed a generally weak genome-wide genetic structure across *P. mandarinus* populations, as supported by the extensive admixture observed in PCA and STRUCTURE analyses (Fig. 2), in line with earlier Sanger-sequencing studies reporting weak genetic differentiation in mitochondrial and nuclear sequences [38, 42, 43]. However, with RADseq, we found that admixture is more pronounced among western and central populations, whereas southeastern populations differ more from the remaining populations. Elevated *F*_ST_ values ( > 0.35) between southeastern and northeastern populations (Figs. 2 and S7) and the association of eastern individuals with spatial predictors in individual-level RDA (Figs. 4, 5, S10) further support localised divergence in this region.

Mantel tests clarified the spatial scale of these patterns. Across all populations, genetic distance showed weak and non-significant correlations with either geographic or environmental distance (Table S6). In contrast, eastern populations exhibited strong and significant IBD and IBE, whereas western populations showed neither of these patterns. However, environmental and geographic distances were themselves highly correlated in the east (*r* = 0.96; Table S6), indicating that geographic isolation and environmental gradients are confounded in this region.

Variance partitioning (Table S3) and piecewise SEM (Fig. 3) provided complementary insights. Geography and environment jointly explained around 10% of the variance in genetic distance, with environment accounting for a larger unique fraction (4.5%) than geography (1.7%; Table S3). The piecewise SEM confirmed that geographic distance significantly predicted environmental distance, reflecting the strong collinearity between spatial and environmental gradients in Taiwan. These results indicate that while neutral spatial processes primarily shape genome-wide structure, environmental heterogeneity, along with geography, also has a detectable influence on genetic divergence.

These results suggest that gene flow dominates genome-wide variation in *P. mandarinus*, but environmental gradients, particularly in topographically complex eastern Taiwan, contribute to localised divergence. This pattern is consistent with theoretical expectations that divergence in this region could be concentrated at a subset of loci under selection, while gene flow or shared ancestry maintains overall genomic homogeneity [101, 102]. Consistent with this interpretation, our GEA analyses identified only a few outlier loci associated with precipitation and temperature gradients (Figs. 4, 5, and S10).

Restricted dispersal may reinforce these regional patterns. Although *P. mandarinus* is capable of flight, its reliance on stream networks for reproduction and larval development may constrain its effective dispersal. While typhoons and extreme weather events periodically alter rainfall and water flow in Taiwan [103, 104], regional hydrological patterns remain relatively stable: Eastern Taiwan, especially the mountain areas, receives nearly twice the annual rainfall of Western Taiwan as indicated in data exploration in several previous studies, e.g. Kao et al. [105] and Nayak et al. [106]; also see Fig. 1 for more recent environmental gradients across Taiwan. Combined with the dense distribution of central and southeastern mountain ranges, this leads to short, steep, and poorly connected river systems’ discharge [103]. Consequently, dispersal events may be restricted between southeastern populations occupying different river systems. In this case, hydrological and landscape topology may contribute more to the observed genetic structure in *P. mandarinus* than ecological factors.

Notably, these geographic patterns were consistent across SNP filtering approaches, confirming they are not artefacts of filtering decisions. HWE and Strict (HWE + LD pruning + MAF) filters resulted in *F*_ST_ values and STRUCTURE patterns comparable to the Full dataset, whereas LD pruning had the strongest effect and inflated *F*_ST_ values. This reflects the potential downward bias in genetic differentiation estimates when loci with large allele frequency differences are disproportionately removed during LD pruning. Nevertheless, across all datasets and under both 70 and 50% completeness, the patterns of the results remained the same: extensive admixture with a gradual latitudinal cline and stronger differentiation of southeastern populations (Supporting Information 2 & 3).

### Phenotypic divergence reveals adaptive signals

Given the weak genome-wide differentiation and limited number of environment-associated loci, we next examined whether phenotypic traits show stronger divergence than expected under neutrality. Our second prediction was that wing traits would show stronger divergence than expected under neutral genetic structure and be associated with environmental gradients. Consistent with this, we observed significant phenotypic divergence in WS and ABA%, with a pronounced latitudinal gradient in ABA% (Table 1). Despite generally weak (though regionally moderate) genomic structure, *P*_ST_ - *F*_ST_ comparisons indicate that phenotypic divergence greatly exceeds neutral genetic differentiation (Table 1), suggesting that these traits are likely shaped by selection rather than genetic drift. However, our GEA analyses using RDA and Gradient Forest analyses, both of which are proven methods for detecting outlier loci [55, 107, 108], identified only a limited number of outlier loci (Figs. 4 and 5). This finding emphasis the contrast between strong phenotypic differentiation and weak genomic signals, suggesting that the selection on single loci alone cannot explain the observed divergence.

The evolutionary history of pigmentation might provide a partial explanation for the observed variation. The apical blackish area (ABA) is shared between species in the genus *Psolodesmus* [38, 109], and pigmented wings may be an ancestral trait in Calopterygid damselflies [48]. Substantial variation in ABA% within and between species [38, 42, 43, 109] despite weak genetic differentiation [38, 42, 43] indicates that trait divergence may result from existing genetic variation, developmental, or environmental influences. More broadly, wing pigmentation and wing size in flying insects have been linked to multiple selective pressures, including thermoregulation, mate signaling, dispersal ability, and predator avoidance [48, 110–112]. To our knowledge, these mechanisms have not yet been tested in *P. mandarinus*, but the association of ABA% and WS with climatic gradients suggests that local thermal or ecological conditions may contribute to divergence in this trait. Future experimental work will be needed to clarify which selective pressures are most relevant.

Several mechanisms could explain the contrast between genetic structure and phenotypic divergence as observed in *P. mandarinus*. First, local adaptation may be shaped by a small number of loci of large effect or by the clustering of adaptive loci, which can maintain localised divergence despite high gene flow [113]. Second, selection may work on standing genetic variation [38, 42], enabling rapid adaptation without new mutations or genome-wide divergence. Third, individual damselflies may develop their wing colours differentially in response to environmental signals through phenotypic plasticity [114], a mechanism increasingly recognised in other taxa experiencing rapid environmental change [115, 116]. Such plasticity in wing traits has also been demonstrated in Odonata and other insects, where developmental temperature and environmental conditions can alter wing size, shape, and pigmentation [117–119]. Nevertheless, studies in Odonata indicate that wing morphology also has a heritable component, indicated by phylogenetic signals [48, 120], suggesting that plasticity and genetic factors may both contribute to shape phenotypic divergence in *P. mandarinus*. While our current data cannot disentangle these mechanisms, future work incorporating common-garden experiments, genome-wide association studies (GWAS), or transcriptomic analyses could help disentangle these possibilities.

### Ecological niches and future range shifts

These trait-based patterns, together with the weak genomic differentiation, suggest that phenotypic variation may translate into ecological divergence under shifting climates. To test our third prediction, we compared the climatic niches and projected distributions of dark- and clear-winged individuals. Our species distribution models predict that the range of *P. mandarinus* will shift toward higher altitudes across different future climate scenarios, particularly in central and eastern Taiwan (Fig. 6 and Table S4). While clear-winged individuals may expand their range, dark-winged individuals may experience range shrinkages due to geographic constraints, such as elevational limits in mountainous areas.

Our findings suggest that environmentally associated phenotypic variations in *P. mandarinus* may shape divergent ecological niches with differing climate sensitivities. While genome-wide structure across populations is weak (Fig. 2), the environmentally associated phenotypic variations indicate potential local adaptation (Table 1). Climate-driven range shifts could, therefore, disrupt these adaptation patterns, leading to a loss of phenotypic diversity and reduced evolutionary resilience [121, 122]. Although recent studies suggest that the outcomes may vary across species, e.g., range shifts may not always result in immediate local extinctions, and the “escalator to extinction” scenario may be less common than previously hypothesised [123, 124], dark-winged individuals remain particularly vulnerable if their suitable habitats become increasingly restricted or inaccessible.

## Conclusion

Our study supports growing evidence that weak genome-wide structure does not preclude meaningful local adaptation [55, 108, 125]. Selection acting on traits such as wing colours and size may enable populations to adapt to regional thermal and hydrological conditions. This suggests that maintaining environmental heterogeneity and connectivity among populations is essential even for species with high dispersal ability. For the conservation of Odonata and other insects relying on shallow freshwater, protecting a mosaic of microclimates and riparian habitats across elevational and latitudinal gradients will help maintain adapted phenotypes and buffer populations against rapid climate change. Our integrative approach also provides a framework for detecting cryptic adaptive variation in less-studied taxa to identify conservation units and management priorities. By linking subtle genetic differences with phenotypic and ecological variation, our study highlights the value of detecting local adaptation in conservation planning, especially for mobile, endemic species facing rapid environmental change.

## Electronic supplementary material

Below is the link to the electronic supplementary material.

Supplementary Material 1

Supplementary Material 2

## Data Availability

Morphological measurement data are provided as Supplementary File 2 with this article. The RADseq data generated and analysed during the current study are available in the NCBI Sequence Read Archive under accession number PRJNA1271275.

## Abbreviations

ABA%: The ratio of apical blackish area of the whole wing (ABA%), one of the wing phenotypic traits that we measured and compared.
GEA analyses: Genotype-environment association analyses
GF: Gradient Forest analysis
HWE: Hardy-Weinberg equilibrium testing
PCNM: Principal Coordinates of Neighbour Matrices analysis, an analysis based on geographic distance matrices, which are calculated from the latitude and longitude coordinates of sampling sites.
RDA: Redundancy analysis, one of the genotype-environment association analyses we applied.
RF: Random Forest analysis
SNP: Single-nucleotide polymorphism
SSP: Shared Socioeconomic Pathways
WS: Wing size, one of the wing phenotypic traits that we measured and compared.
