# Supplementary material for: Detecting local adaptation under weak genetic structure in an endemic damselfly: an integrative eco-evolutionary approach
Source: BMC Ecol Evol. 2026 Feb 2;26:2. doi: 10.1186/s12862-025-02462-z (PMC12862917; doi:10.1186/s12862-025-02462-z)
Supplement: Supplementary file 1 — Supplementary Material 1 [file 12862_2025_2462_MOESM1_ESM.docx]

# Supporting Information 1. Supporting Tables and Figures mentioned in the main text.

*Table S1.* The 19 bioclimatic variables from CHELSA. While all variables were included in initial models, we marked those retained for downstream analyses after sequential filtering with "V"

| **Bioclimatic variables** | **Abbrev.** | **Dark-winged** | **Clear-winged** | **All populations** |
| --- | --- | --- | --- | --- |
| Annual Mean Temperature | bio 1 |  |  |  |
| Mean Diurnal Range | bio 2 |  |  |  |
| Isothermality | bio 3 | V | V | V |
| Temperature Seasonality | bio 4 | V | V | V |
| Max Temperature of Warmest Month | bio 5 |  |  |  |
| Min Temperature of Coldest Month | bio 6 |  |  | V |
| Temperature Annual Range | bio 7 | V | V | V |
| Mean Temperature of Wettest Quarter | bio 8 | V |  |  |
| Mean Temperature of Driest Quarter | bio 9 |  |  |  |
| Mean Temperature of Warmest Quarter | bio 10 |  | V | V |
| Mean Temperature of Coldest Quarter | bio 11 |  |  |  |
| Annual Precipitation | bio 12 |  | V | V |
| Precipitation of Wettest Month | bio 13 |  |  | V |
| Precipitation of Driest Month | bio 14 |  |  |  |
| Precipitation Seasonality | bio 15 |  | V | V |
| Precipitation of Wettest Quarter | bio 16 | V |  |  |
| Precipitation of Driest Quarter | bio 17 | V |  | V |
| Precipitation of Warmest Quarter | bio 18 |  |  | V |
| Precipitation of Coldest Quarter | bio 19 |  |  |  |

Table S2. Number of specimens applied for genomic analysis (N_G_) and phenotypic analysis (N_P_).

| Sample site | *N*_G_ | *N*_P_ |
| --- | --- | --- |
| Alibang | 6 | 6 |
| Daxi | 13 | 13 |
| Longtan | 11 | 11 |
| Guanxi | 12 | 12 |
| Neiwan | 5 | 5 |
| Miaoli | 5 | 4 |
| Lianhuachi | 28 | 28 |
| Maolin | 7 | 7 |
| Shizi | 8 | 8 |
| Nangang | 9 | 9 |
| Fushan | 27 | 24 |
| Xiulin | 6 | 6 |
| Haiduan | 4 | 4 |
| Total | 141 | 137 |

*Note: Sampling sites are ordered by location from north to south and west to east.*

*Table S3.* Results of variance partitioning analysis following the redundancy analysis (RDA). Partitioning of variance is explained by geographical (geo.) and environmental (env.) factors. The table shows the percentage of variance explained (PVE%) by combined and individual fractions, and *F*-statistics were used to assess the significance of each fraction, with statistical significance indicated as: *p* < 0.05 (*), *p* < 0.01 (**), *p* < 0.001 (***).

| Combined fractions | PVE(%) |
| --- | --- |
| F~geo. | 5.42*** |
| F~env. | 8.28*** |
| Individual fractions |  |
| F~geo.\| env. | 1.66*** |
| F~env.\| geo. | 4.52*** |
| Total explained | 9.94*** |
| Total unexplained | 90.06 |
| Total | 100 |

Table S4. The change of potential distribution range under different future Shared Socioeconomic Pathways (SSPs). Positive values indicate an increase, while negative values indicate a decrease in the future potential distribution range.

| Scenario | SSP126 | | | SSP370 | | | | SSP585 | | | |  |
| --- | --- | --- | --- | --- | --- | --- | --- | --- | --- | --- | --- | --- |
| Period | 2011-2040 | 2041-2070 | 2071-2100 | | 2011-2040 | 2041-2070 | 2071-2100 | | 2011-2040 | 2041-2070 | 2071-2100 | |
| Dark-winged | 48.5% | 24.9% | 46.1% | | -4.4% | -5.9% | -22.9% | | 42.4% | 39.5% | 37.3% | |
| Clear-winged | 54.8% | 76.6% | 102.3% | | 76.7% | 58.5% | 41.1% | | 65.4% | 23.7% | 50.8% | |
| All Populations | 39.2% | 14.1% | 120.6% | | 22.0% | -9.8% | 46.0% | | 40.1% | 81.9% | 105.6% | |


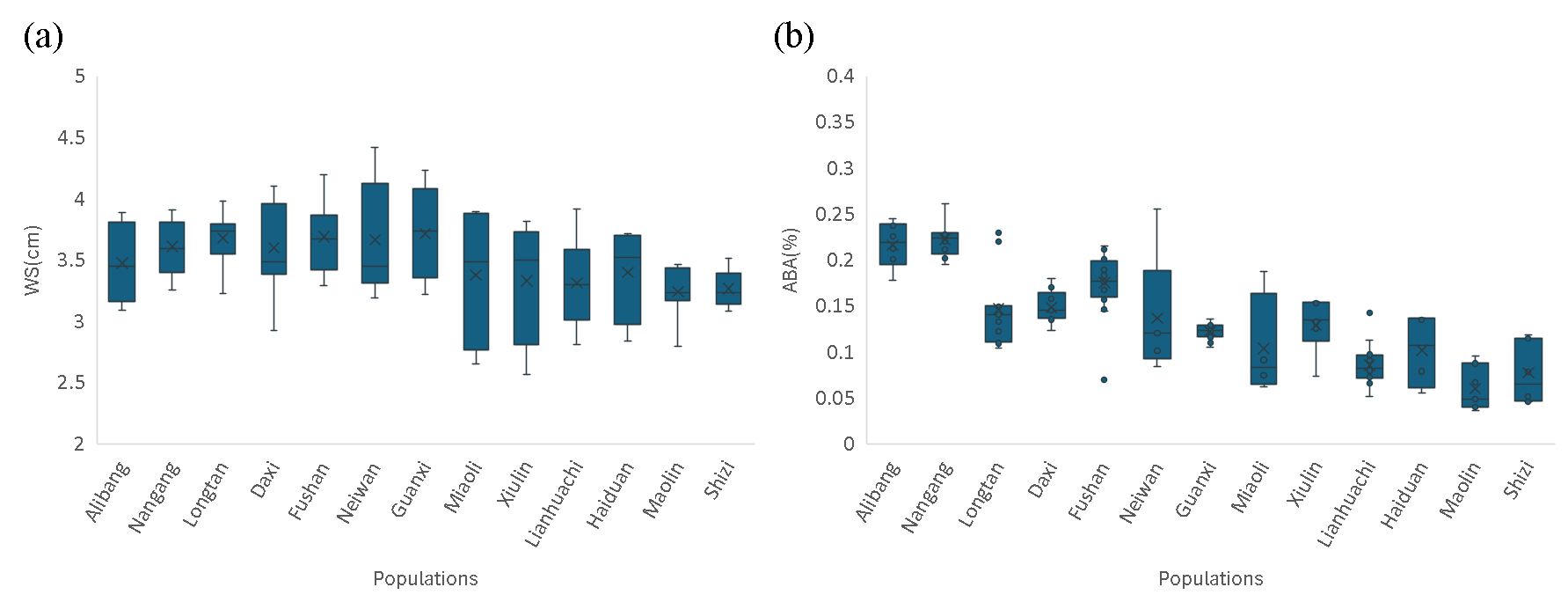


Figure S1. Phenotypic variations of (a) wing size (WS) and (b) the ratio of the apical blackish area of the whole wing (ABA%) are different among populations (WS, F = 3.34, p <0.001; ABA%, F= 24.61, p <0.001).


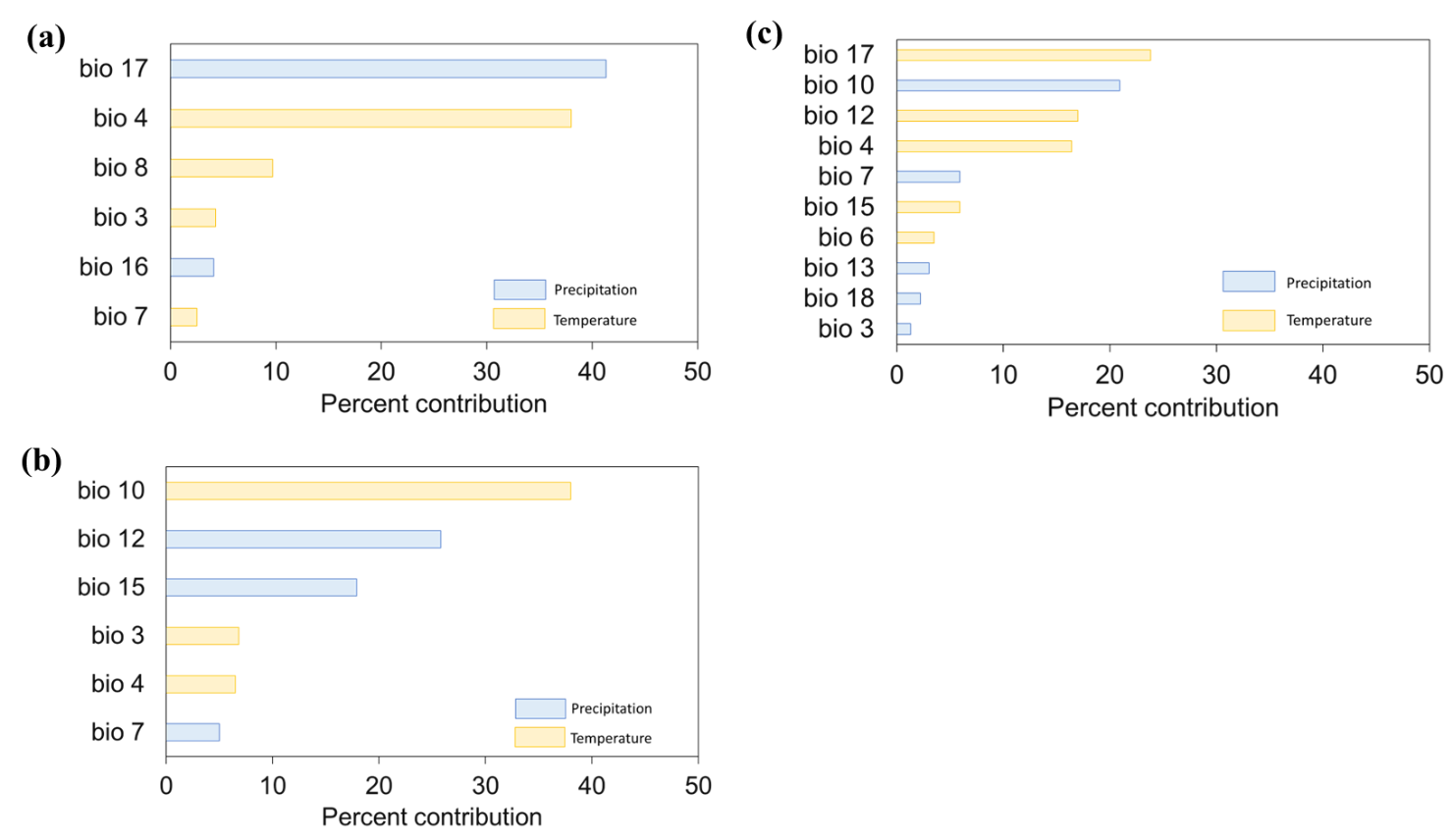


*Figure S2.* The variable contribution of distribution models for (a) Dark-winged individuals, (b) Clear-winged individuals and (c) all individuals.


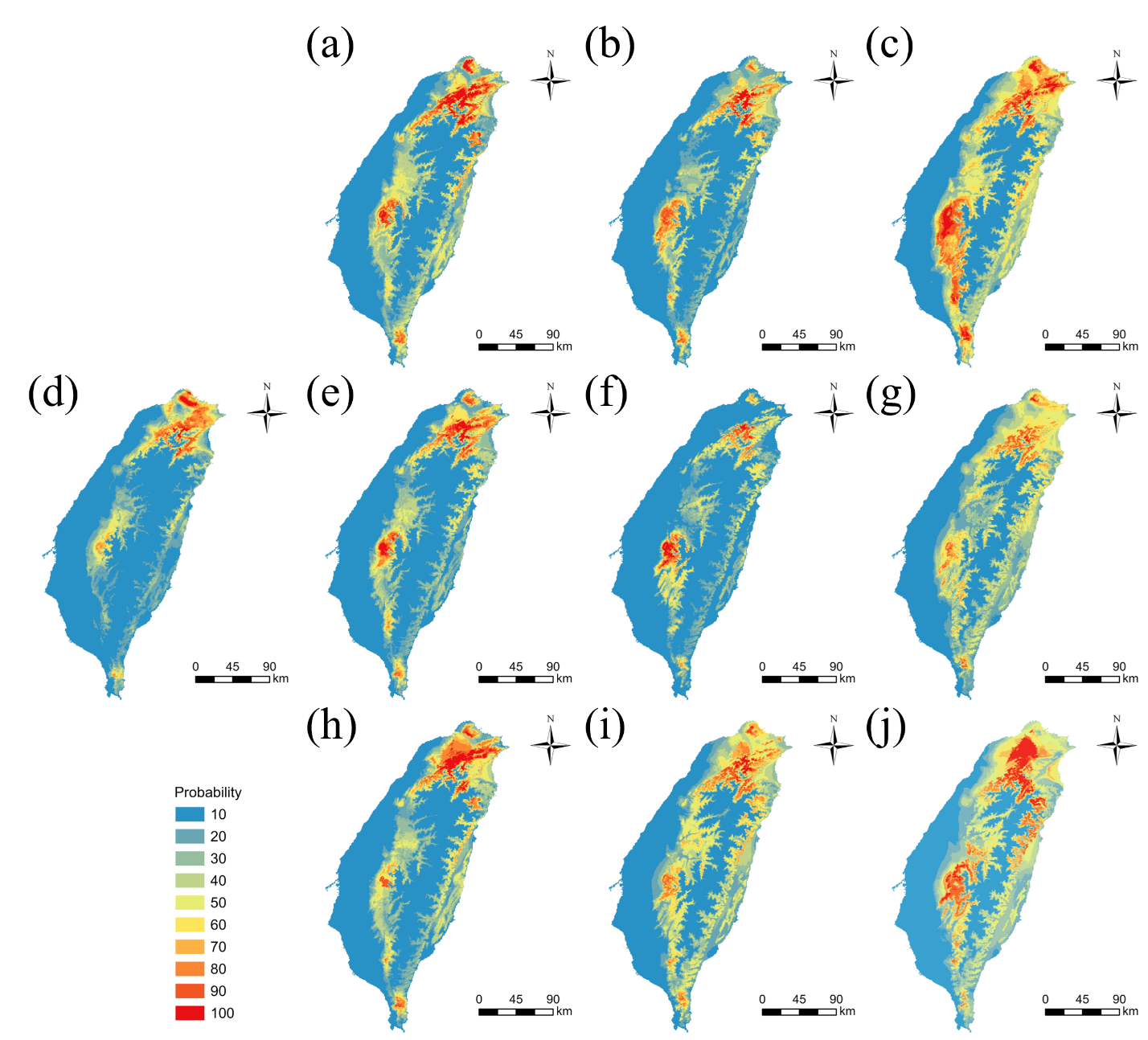


*Figure S3.* Projected distribution of *Psolodesmus mandarinus* (jointly with all individuals regardless of phenotypes) under current time (d); future distribution under SSP126 in (a) 2011-2040, (b) 2041-2070, and (c) 2071-2100; (d) current time; future distribution under SSP370 in (e) 2011-2040, (f) 2041-2070, and (g) 2071-2100; and future distribution under SSP585 in (h) 2011-2040, (i) 2041-2070, and (j) 2071-2100.


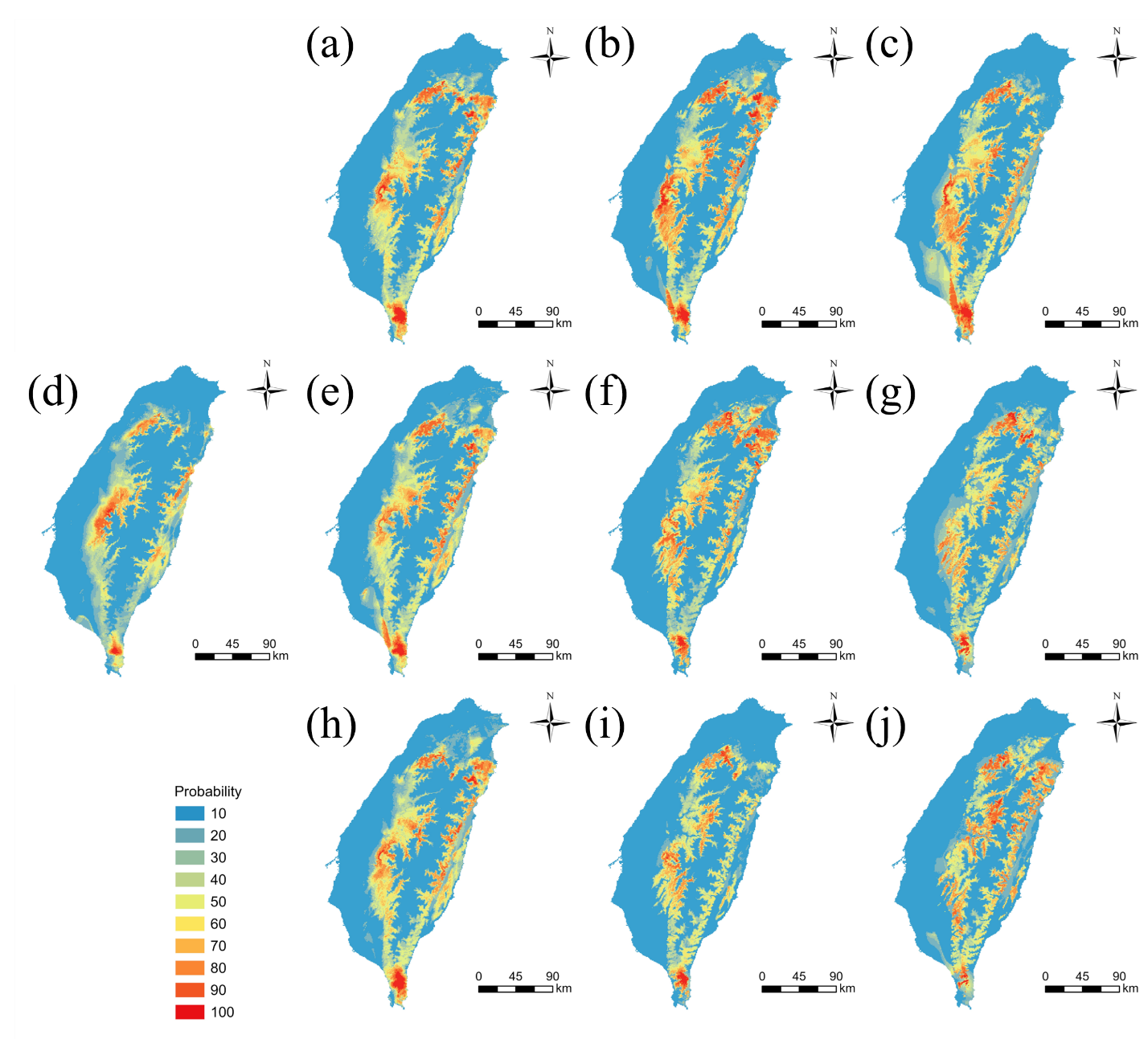


*Figure S4.* Projected distribution of clear-winged individuals of *Psolosdesmus mandarinus* under current time (d); future distribution under SSP126 in (a) 2011-2040, (b) 2041-2070, and (c) 2071-2100; (d) current time; future distribution under SSP370 in (e) 2011-2040, (f) 2041-2070, and (g) 2071-2100; and future distribution under SSP585 in (h) 2011-2040, (i) 2041-2070, and (j) 2071-2100.


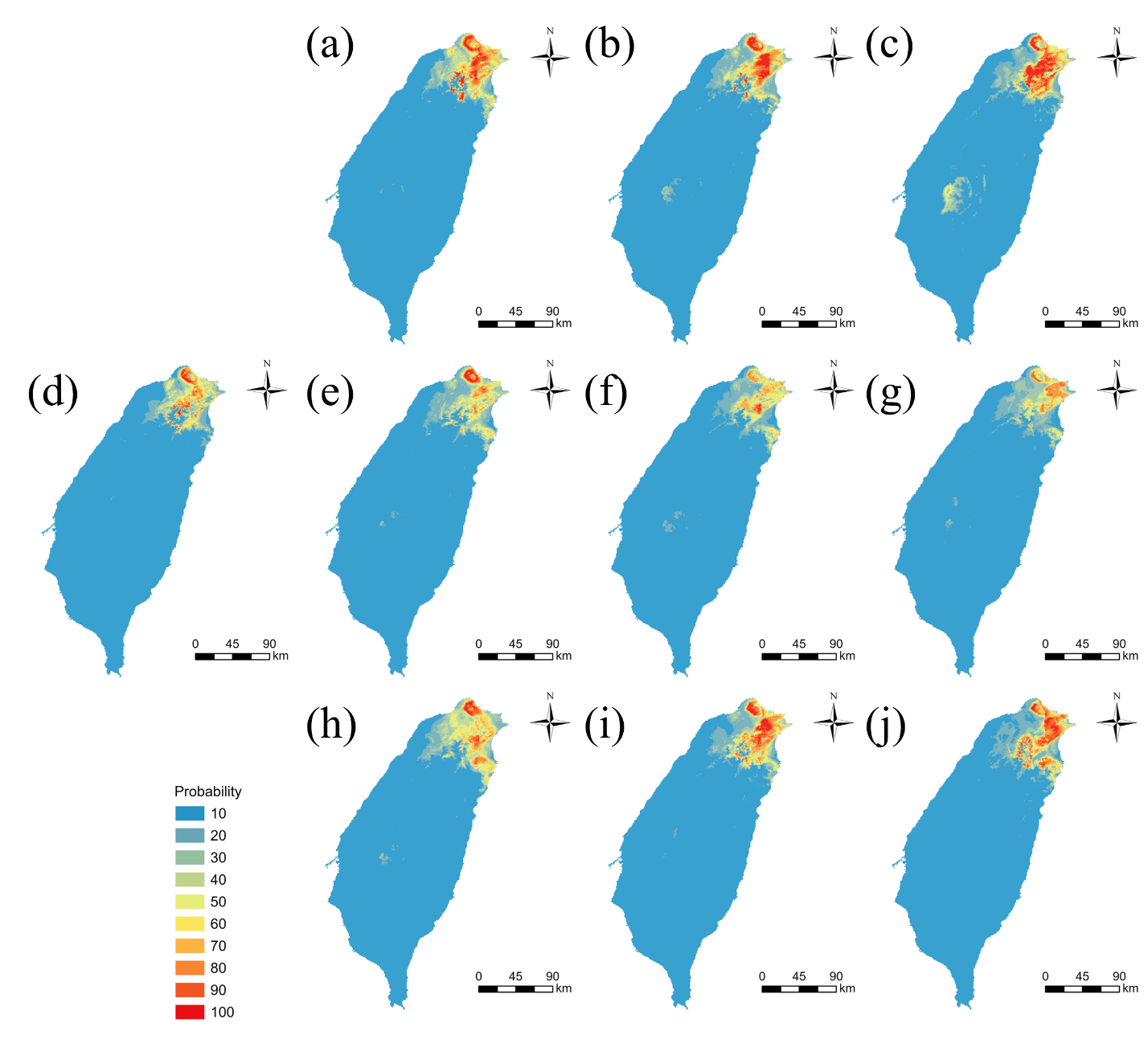


*Figure S5.* Projected distribution of dark-winged individuals of *Psolodesmus mandarinus* under current time (d); future distribution under SSP126 in (a) 2011-2040, (b) 2041-2070, and (c) 2071-2100; (d) current time; future distribution under SSP370 in (e) 2011-2040, (f) 2041-2070, and (g) 2071-2100; and future distribution under SSP585 in (h) 2011-2040, (i) 2041-2070, and (j) 2071-2100.

# Supporting Information 2. Additional analyses with filtered SNP datasets.

## Introduction and Methods

In the main text, analyses were conducted using the complete SNP dataset to maximise coverage and retain weak signals of genetic differentiation [1, 2]. However, non-filtered datasets may also include errors from genotyping and sequencing, which could bias inference [3, 4]. To evaluate the robustness of our conclusions, we generated conservatively filtered datasets using PLINK v1.9 [5].

HWE was calculated separately for each population, and loci deviating from HWE were removed under two criteria: (i) deviations in any single population (“Out Any”), and (ii) deviations consistently observed across all populations (“Out All”)[6]. The Out All criterion did not exclude any loci and thus was equivalent to the full SNP dataset, whereas the Out Any criterion removed a subset of loci (Table S5). Because deviations from HWE can arise both from technical artefacts (e.g., genotyping errors) and biological processes (e.g., population structure, selection) [6], applying HWE filters can reduce false signals but may also remove informative loci.

Downstream analyses were therefore conducted using four datasets (Table S5):

1. Full – no additional filtering, the same as the dataset used in the main text;
2. LD+MAF - LD pruning (r² > 0.5) and MAF filtering (< 0.01), no HWE filtering;
3. HWE – HWE Out Any filtering only;
4. Strict – All three filters applied (HWE Out Any, LD pruning and MAF filtering).

Population structure analyses and Mantel tests were repeated across all four datasets. Genotype–environment association (GEA) analyses (RDA, Gradient Forest) and phenotype-environment association analysis (Random Forest) were repeated only with the LD+MAF dataset to avoid the potential Wahlund effect [7](Table S5). Results from these filtered datasets are presented here in comparison with the unfiltered results in the main text (the same as the Full dataset here) to assess the robustness of our findings.

## Results and Discussion

Across all four SNP datasets with different filtering approaches (Full, LD+MAF, HWE, and Strict), population structure analyses consistently showed weak differentiation and high admixture. STRUCTURE supported *K* = 6 in the Full dataset and *K* = 3 in the filtered datasets (Figure S6), but in all cases, populations showed partial clustering rather than discrete groups, with only the southeastern populations (Xiulin, Haiduan, Shizi) showing clearer separation. Pairwise *F*_ST_ estimates supported this pattern: southeastern populations were consistently the most differentiated, whereas northern and central populations showed very low divergence (Figure S7). The HWE and Strict datasets resulted in highly similar *F*_ST_ values with the Full dataset, while LD+MAF filtering inflated *F*_ST_ overall and caused larger shifts among comparisons, particularly involving Alibang and central populations. PCA and Procrustes PCA also revealed continuous gradients without sharp breaks between populations (Figures S8 & S9). Mantel tests indicated robust isolation by distance (IBD) but no significant isolation by environment (IBE), with results showing the same directions across datasets (Table S6). These results demonstrate that despite quantitative differences among filtering approaches, all methods converged on the same qualitative outcome: a gradual latitudinal cline with relatively stronger differentiation of southeastern populations.

Analyses of environmental associations showed consistent results across datasets. Using the LD+MAF dataset, we identified broadly similar predictors to those from the Full dataset, with both spatial (PCNM axes) and climatic variables contributing strongly (Figure S10). Although the ranking of climatic predictors differed (precipitation of the driest quarter was among the top variables in the Full dataset but less prominent in the LD+MAF dataset, where precipitation seasonality and temperature-related variables gained relative importance), both datasets consistently highlighted precipitation- and temperature-related gradients as the major environmental drivers of genetic variation. Phenotype–environment associations also highlighted precipitation and temperature variables as the strongest predictors across RDA, Gradient Forest, and Random Forest analyses (Figure S11). Despite shifts in the relative importance of individual variables, the overlap in key environmental drivers indicates that both genetic and phenotypic associations are robust to SNP filtering.

In summary, although the relative ranking of individual predictors varied, both the Full and filtered datasets converged on the same conclusion reported in the main text: weak genome-wide differentiation, accompanied by consistent signals of local adaptation, was shaped primarily by precipitation and temperature gradients.

Table S5. Summary of SNP datasets and filtering criteria used for robustness analyses.

| Dataset | *N*_loci_ included under different completeness thresholds | | Filter applied | | | Analyses Conducted | | | |
| --- | --- | --- | --- | --- | --- | --- | --- | --- | --- |
|  | 70% | 50% | LD  (r²>0.5) | MAF (<0.01) | HWE  (Out Any) | Population structure (Structure, *F*_ST_, conventional PCA, Procrustes PCA) | Mantel test for IBE and IBD analyses | GEA analyses and  (only with 70% completeness) | Phenotype-association analysis (only with 70% completeness) |
| Full* | 656 | 13,857 | - | - | - | ✓ | ✓ | ✓ | ✓ |
| LD+MAF | 265 | 3,455 | + | + | - | ✓ | ✓ | ✓ | ✓ |
| HWE | 592 | 13,238 | - | - | + | ✓ | ✓ | - | - |
| Strict | 219 | 3,200 | + | + | + | ✓ | ✓ | - | - |

*
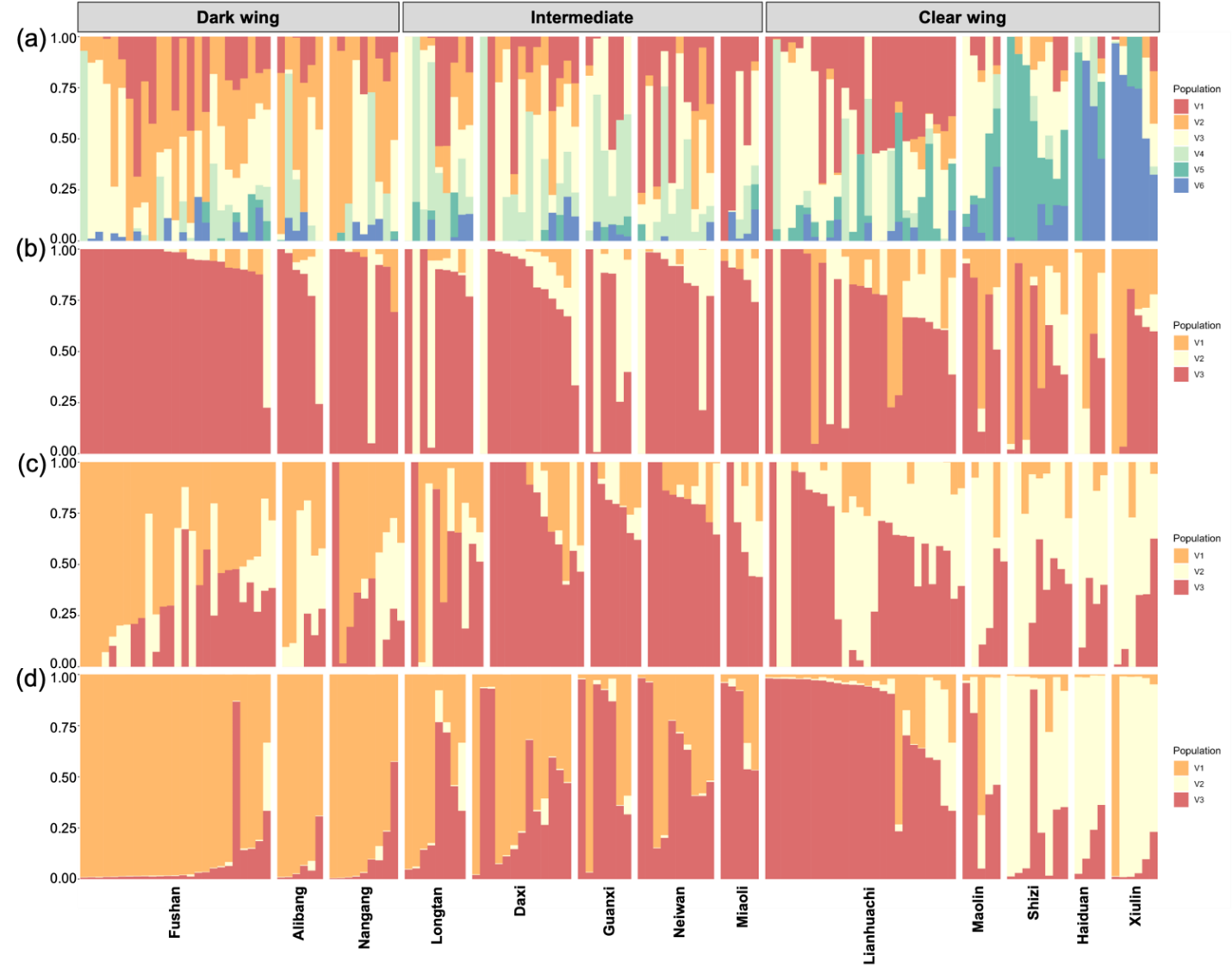
*

*Figure S6.* Results of STRUCTURE with the optimal number of *K* for each of the four filtering datasets with 70% completeness: (a) Full, (b) LD+MAF, (c) HWE, and (d) Strict.


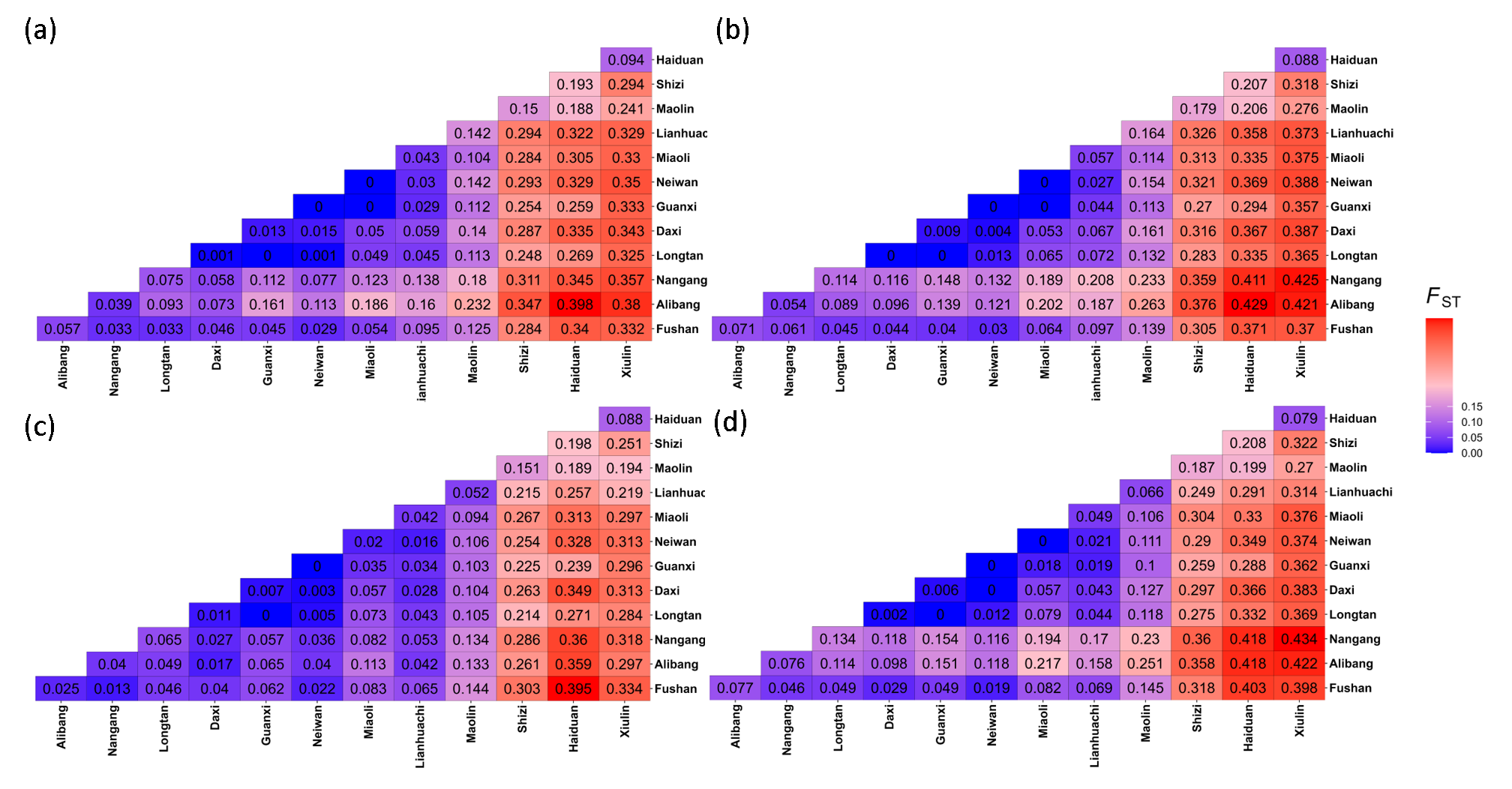


*Figure S7*. Pairwise *F*_ST_ heatmaps based on the four filtering datasets with 70% completeness: (a) Full, (b) LD+MAF, (c) HWE, and (d) Strict.


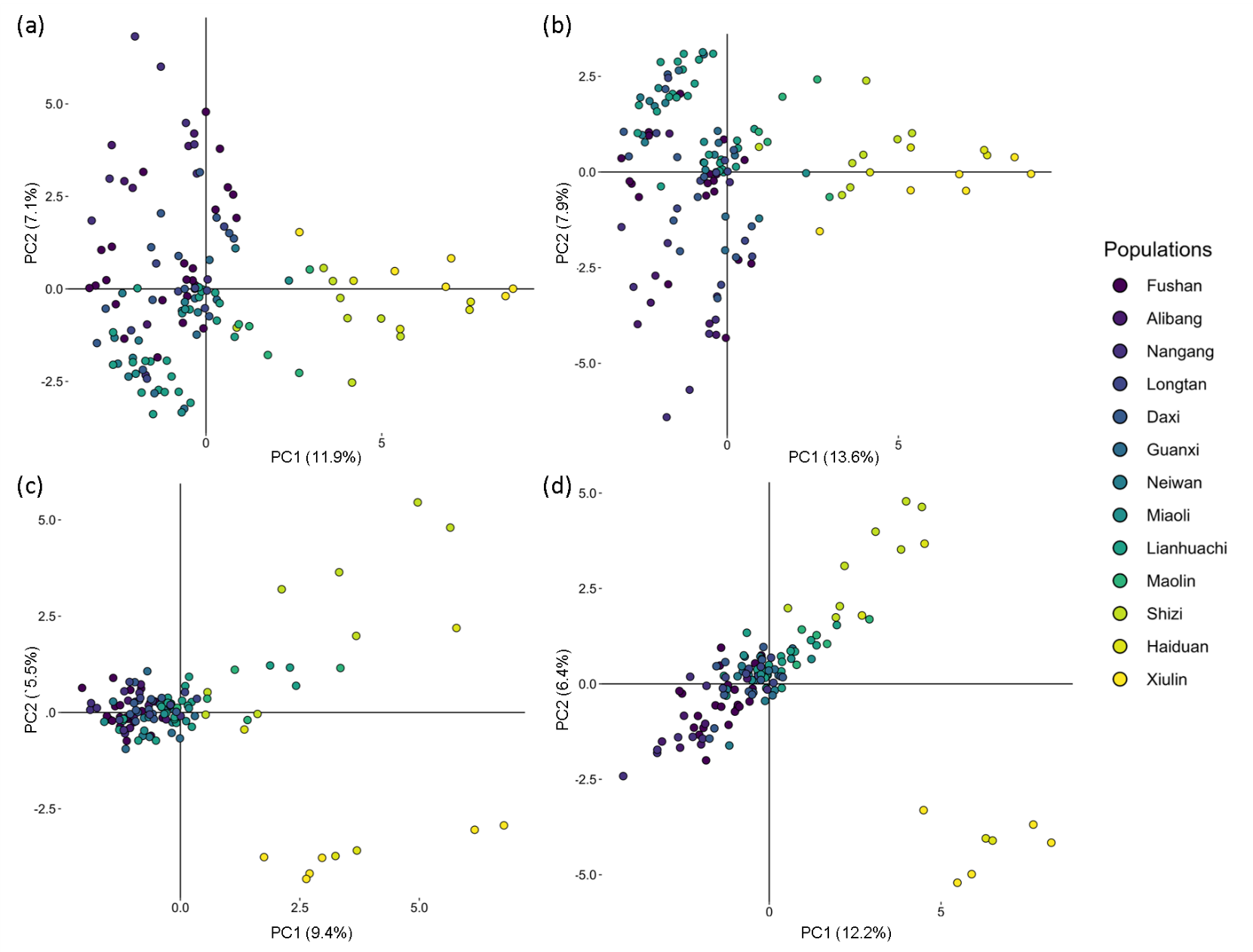


*Figure S8.* Results of the conventional Principal Component Analysis (PCA) based on the four filtering datasets with 70% completeness: (a) Full, (b) LD+MAF, (c) HWE, and (d) Strict.


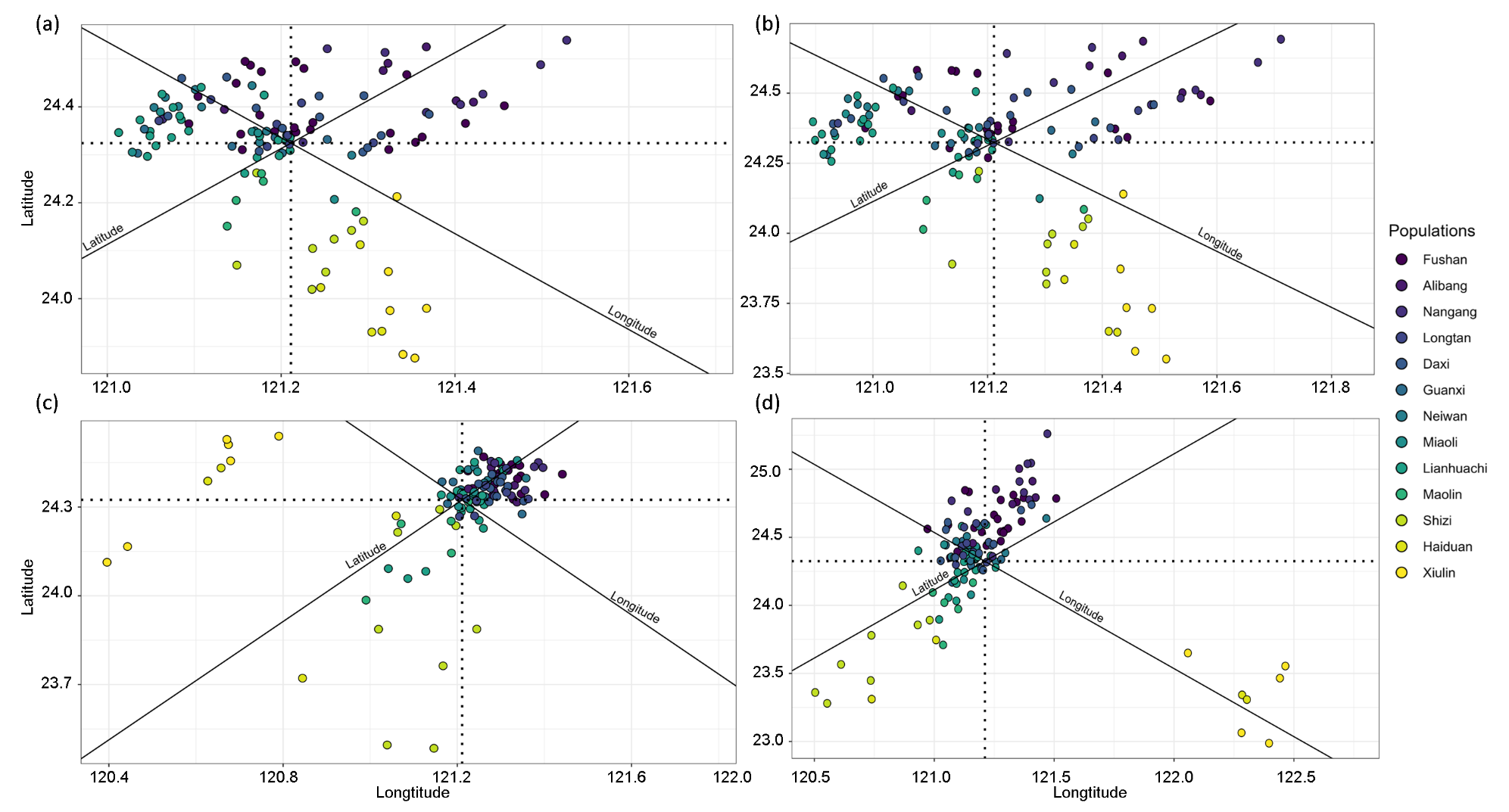


*Figure S9*. Results of the Procrustes Principal Component Analysis (Procrustes PCA) based on the four filtering datasets with 70% completeness: (a) Full, (b) LD+MAF, (c) HWE, and (d) Strict.

|  |  | Full | | | LD+MAF | | | HWE | | | Strict | | |
| --- | --- | --- | --- | --- | --- | --- | --- | --- | --- | --- | --- | --- | --- |
| Var1 | Var2 | All | East | West | All | East | West | All | East | West | All | East | West |
| Dgen | Dgeo | 0.21 | 0.70 | -0.28 | 0.24 | 0.70 | -0.37 | 0.15 | 0.74 | -0.26 | 0.15 | 0.74 | -0.26 |
| Dgen | Denv | 0.02 | 0.79* | 0.04 | 0.01 | 0.75* | -0.10 | 0.07 | 0.79* | -0.06 | 0.07 | 0.79* | -0.06 |
| Denv | Dgeo | -0.22 | 0.96* | -0.24 | -0.22 | 0.97* | -0.15 | -0.22 | 0.97* | -0.24 | -0.22 | 0.97 | -0.24 |

*Table S6*. Results of the Mantel test correlations among genetic distance (Dgen), geographic distance (Dgeo), and environmental distance (Denv) under the four filtering datasets with 70% completeness: Full, LD+MAF, HWE, and Strict. Correlations are reported for all populations (All), eastern populations (East), and western populations (West). Significant correlations (*p* < 0.05) are indicated with an asterisk (*).


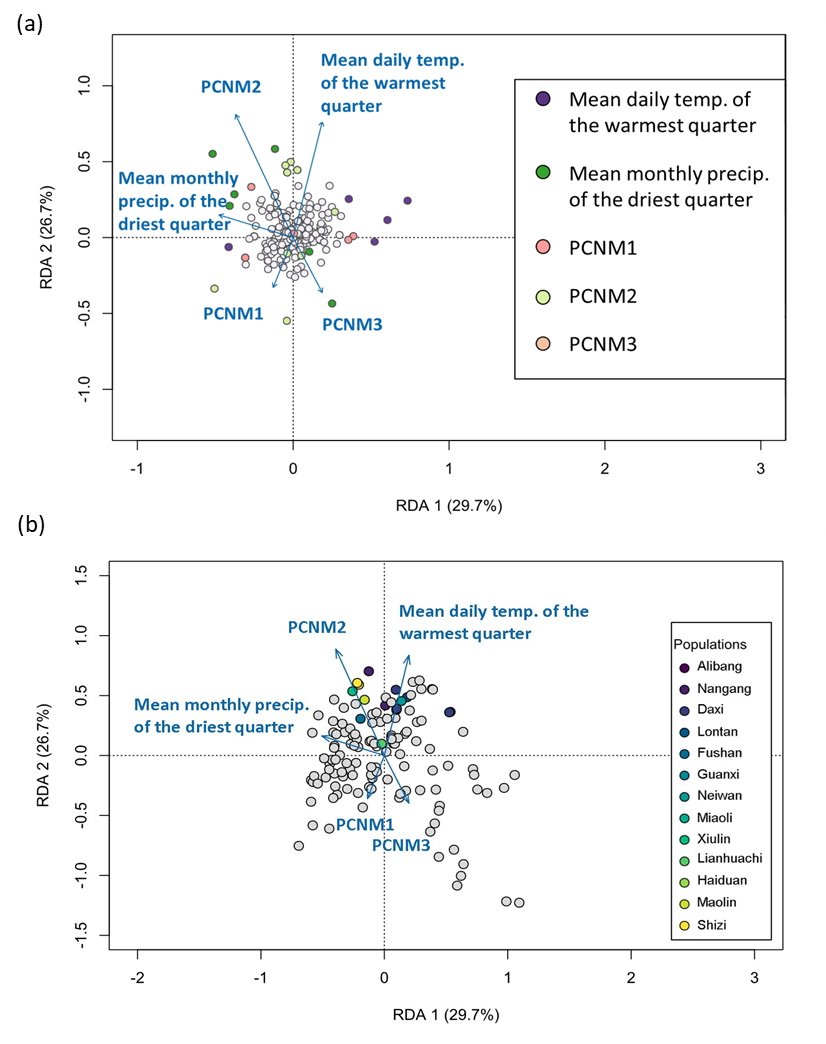


*Figure S10*. Results of Redundancy analysis (RDA) of genetic variation in *Psolodesmus mandarinus* based on the LD+MAF dataset with 70% completeness, showing the relationship with environmental and spatial predictors. Panel (a) presents SNP loadings, and panel (b) displays individual scores. Arrows represent the direction and strength of correlations between predictor variables and the RDA axes; longer arrows indicate stronger associations. In (a), colored points denote SNPs most strongly associated with specific predictors, while in (b), colored points represent individuals from different populations most strongly associated with particular environmental or spatial variables. Predictor variables include climatic factors (e.g., mean daily temperature of the warmest quarter, mean monthly precipitation of the driest quarter) and spatial components derived from PCNM analysis (PCNM1–3).


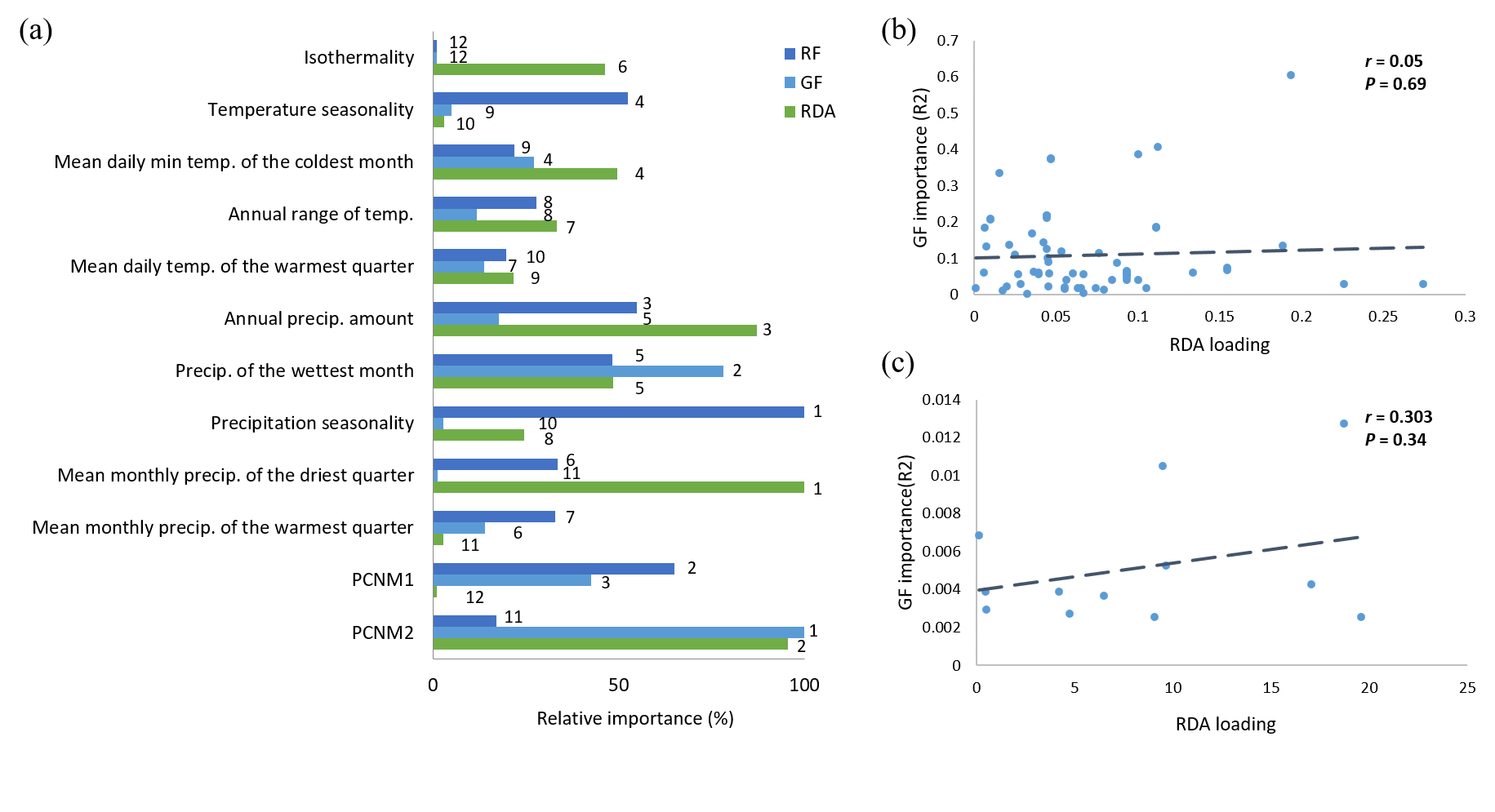


*Figure S11*. Comparison of environmental predictor importance across methods based on LD+MAF dataset with 70% completeness. (a) Relative importance (%) of environmental predictors from Redundancy Analysis (RDA), Gradient Forest (GF), and Random Forest (RF), with numeric ranks indicated beside the bars. (b) Relationship between RDA loadings and GF importance for SNP loci. (c) Relationship between RDA loadings and GF importance for environmental predictors. In (b) and (c), each point represents an SNP or predictor, respectively; dashed lines show regression trends in both.

# Supporting Information 3. Robustness to Completeness Threshold

## **Introduction and Methods**

In the main text and Supplementary Information 2, all SNP-based analyses were conducted using a 70% locus completeness threshold, which is widely applied in RADseq studies to balance marker retention and data quality. To test whether our conclusions are sensitive to this threshold, we repeated population structure analyses under a more relaxed 50% completeness threshold. This dataset retained 13,857 loci prior to filtering, compared to 656 loci at the 70% threshold. Analyses repeated those performed with the 70% dataset, including STRUCTURE, PCA, and the Mantel test. Filtering schemes (HWE “Out Any”, LD pruning, and MAF < 0.01) were applied as in Supporting Information 2.

## **Results and Discussion**

As already shown in Table S5, relaxing the completeness threshold successfully retained more SNP loci (13,857 loci retained before filtering). STRUCTURE consistently supported weak genome-wide differentiation and high admixture under the 50% threshold (Figure S12). The optimal *K* was 3 across all four filtering approaches. Pairwise *F*_ST_ values were generally moderate to low (0.05-0.20 for most population pairs), with the strongest differentiation observed between southeastern and northern populations (Figure S13). PCA and Procrustes PCA reported the same pattern of weak structure with a latitudinal gradient, but clustering was more diffuse and variance explained was lower (PC1 ≈ 8.4–9.5%, PC2 ≈ 4.2–4.9; Figures S14–S15) than in the 70% datasets (PC1 ≈ 11.9–13.6%, PC2 ≈ 5.5–7.9; Figures S8-S9). Mantel tests also showed weak overall IBD/IBE, with stronger signals in eastern populations (Table S8).

Both 50% and 70% thresholds support the same qualitative conclusions: weak genome-wide structure, extensive admixture, a latitudinal cline, and relatively greater divergence of southeastern populations. However, the 50% dataset produced lower *F*_ST_ and weaker PCA separation than the 70% dataset (which explains more variance; PC1 ≈ 11.9–13.6%, PC2 ≈ 5.5–7.9). These differences are most plausibly due to the higher proportion of missing data in the 50% set, which can dilute differentiation signals and reduce stability in multivariate analyses. Therefore, we present results of the main analyses on the 70% completeness Full dataset in the main text, which shows more conservative and stable estimates across analyses, and present the 50% results here as a robustness check, showing that the qualitative inferences are consistent.


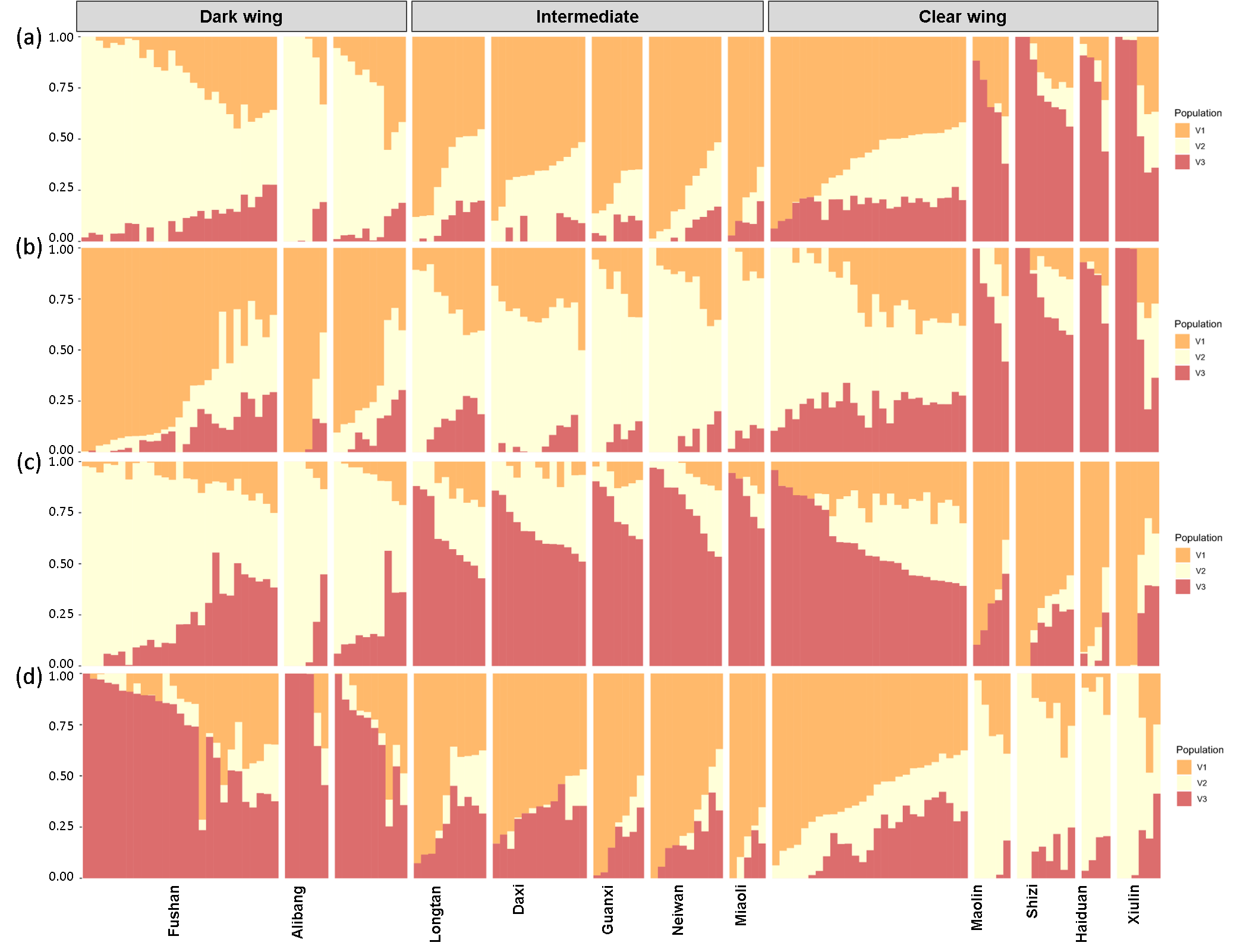


*Figure S12*. Results of STRUCTURE with the optimal number of *K* for each of the four filtering datasets with 50% completeness: (a) Full, (b) LD+MAF, (c) HWE, and (d) Strict.


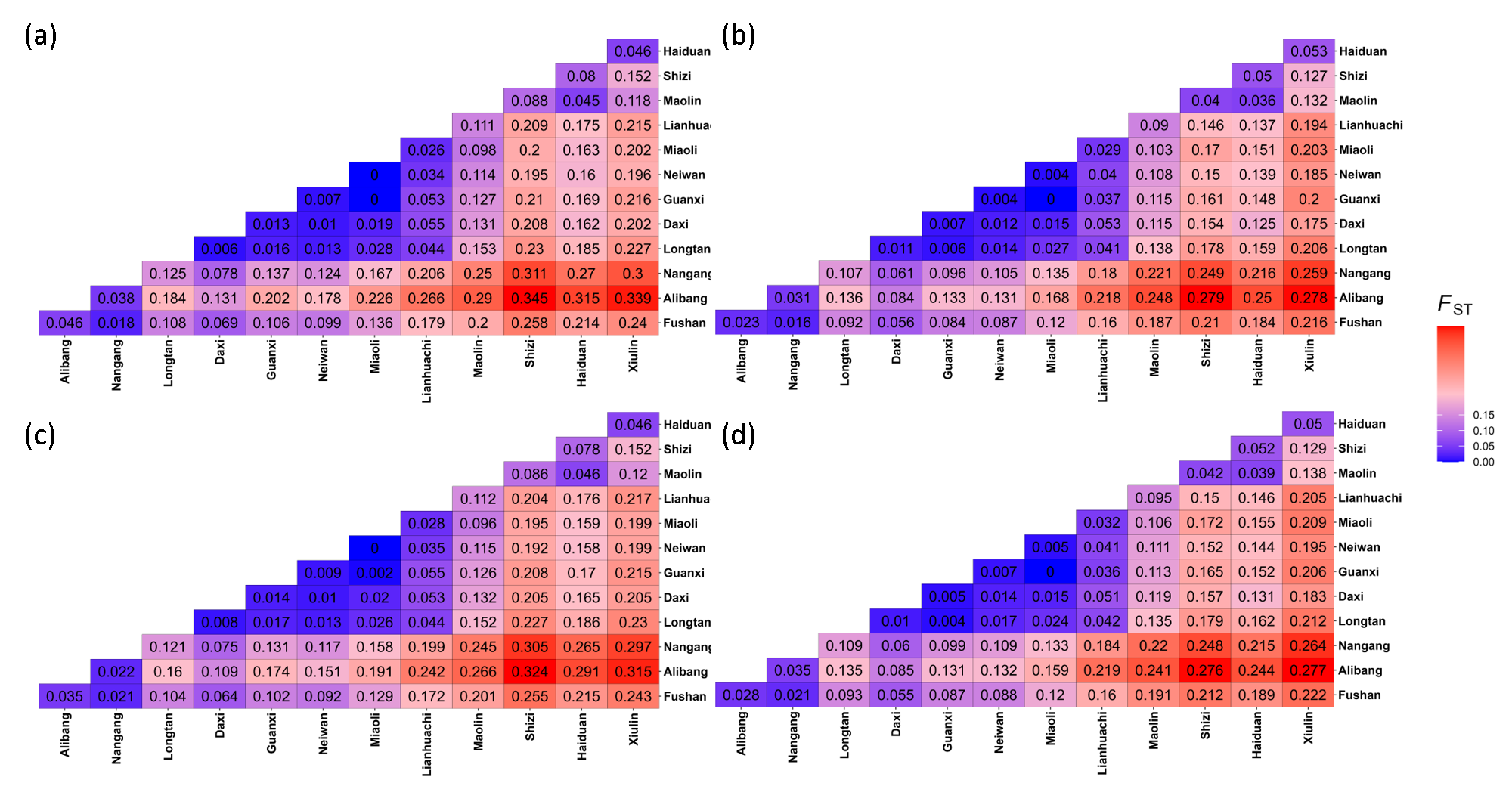


*Figure S13*. Pairwise *F*_ST_ heatmaps based on the four filtering datasets with 50% completeness: (a) Full, (b) LD+MAF, (c) HWE, and (d) Strict.


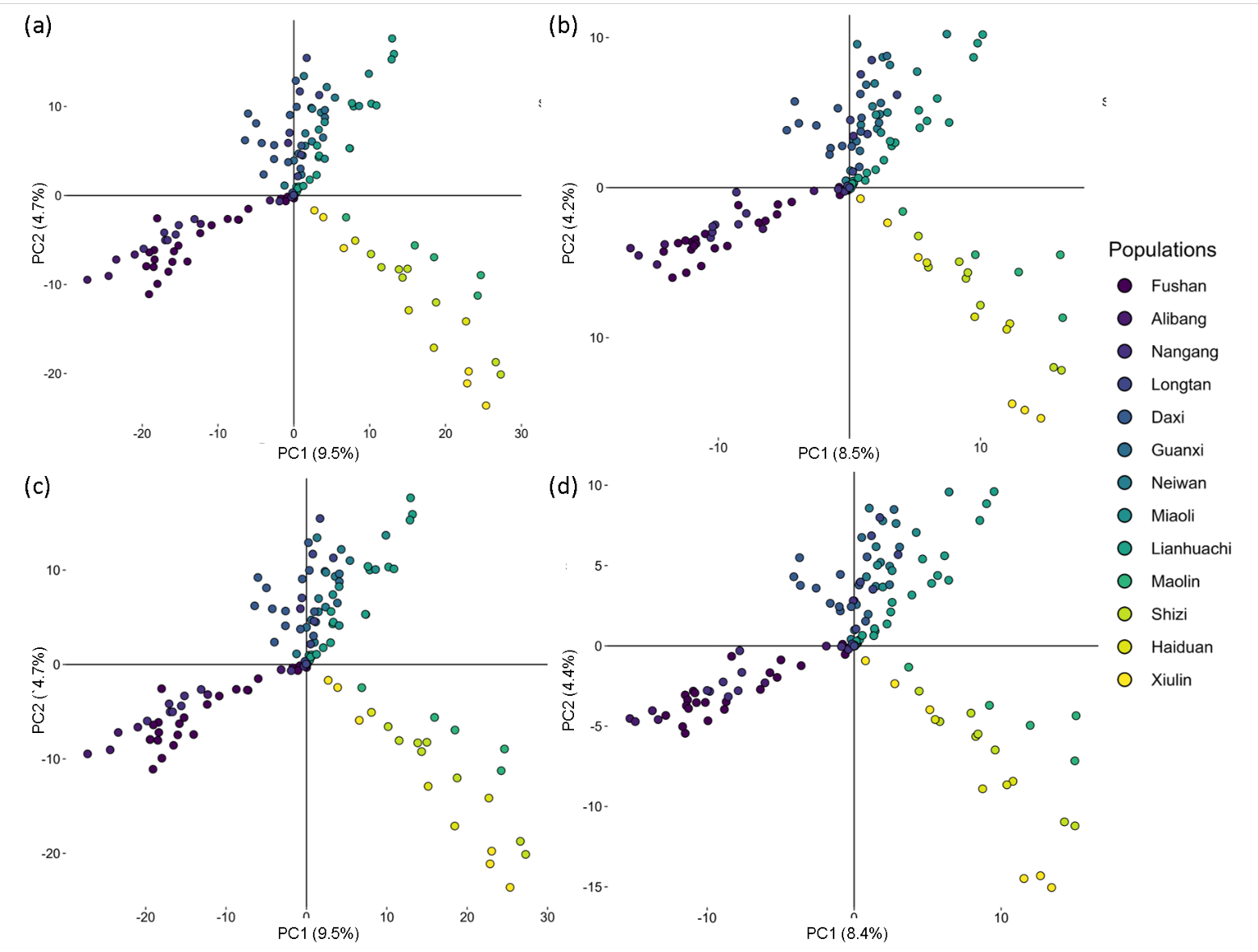


*Figure S14*. Results of the conventional Principal Component Analysis (PCA) based on the four filtering datasets with 50% completeness: (a) Full, (b) LD+MAF, (c) HWE, and (d) Strict.


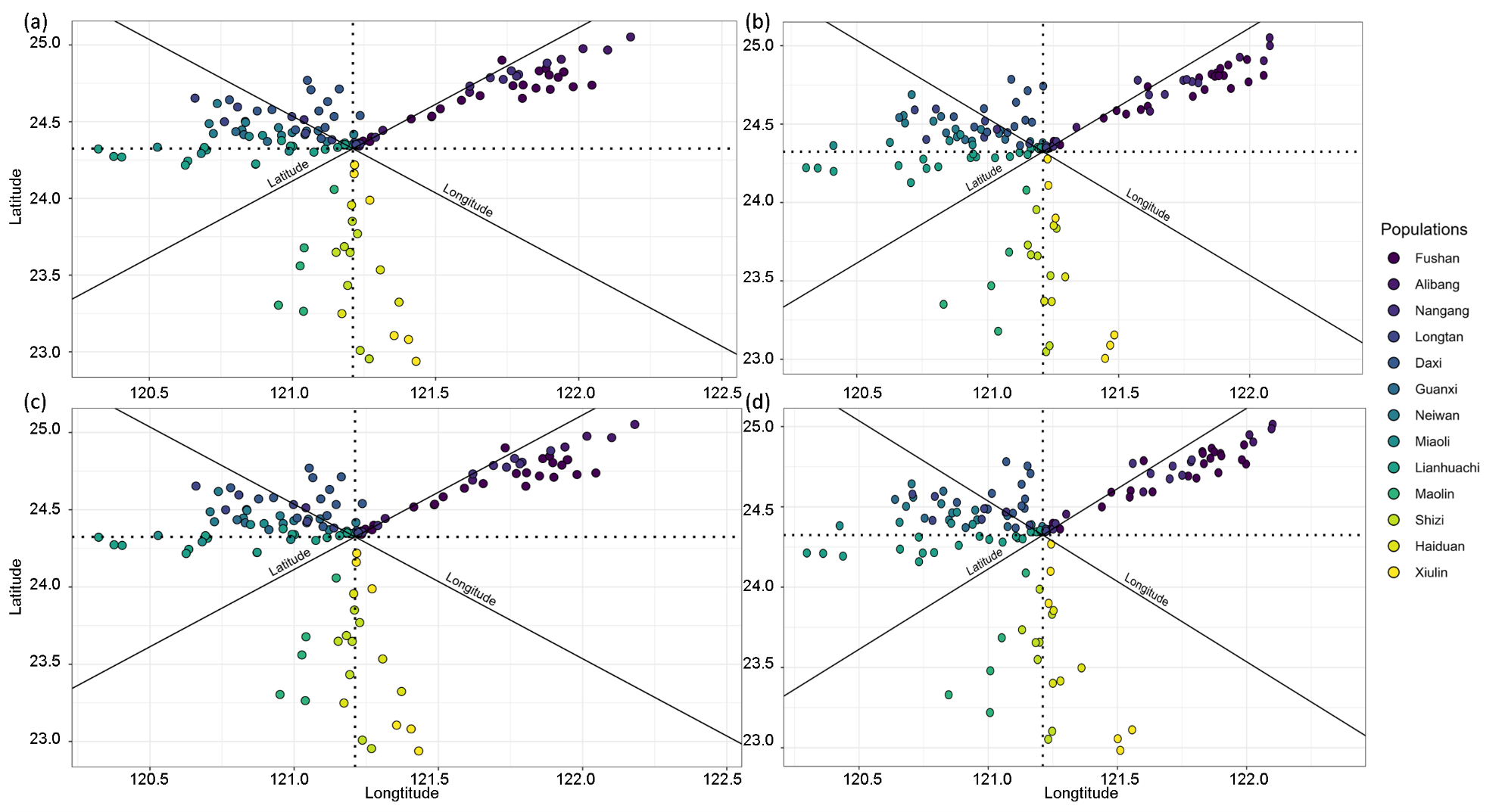


*Figure S15*. Results of the Procrustes Principal Component Analysis (Procrustes PCA) based on the four filtering datasets with 50% completeness: (a) Full, (b) LD+MAF, (c) HWE, and (d) Strict.

*Table S7*. Results of the Mantel test correlations among genetic distance (Dgen), geographic distance (Dgeo), and environmental distance (Denv) under the four filtering datasets with 50% completeness: Full, LD+MAF, HWE, and Strict. Correlations are reported for all populations (All), eastern populations (East), and western populations (West). Significant correlations (*p* < 0.05) are indicated with an asterisk (*).

|  |  | Full | | | LD+MAF | | | HWE | | | Strict | | |
| --- | --- | --- | --- | --- | --- | --- | --- | --- | --- | --- | --- | --- | --- |
| Var1 | Var2 | All | East | West | All | East | West | All | East | West | All | East | West |
| Dgen | Dgeo | 0.20 | 0.63 | -0.26 | 0.21 | 0.63 | -0.26 | 0.15 | 0.67 | -0.25 | 0.16 | 0.67 | -0.25 |
| Dgen | Denv | -0.09 | 0.62 | -0.11 | -0.09 | 0.63 | -0.11 | -0.08 | 0.70 | -0.06 | -0.08 | 0.70 | -0.06 |
| Denv | Dgeo | -0.22 | 0.97* | -0.15 | -0.22 | 0.97* | -0.15 | -0.22 | 0.97* | -0.16 | -0.22 | 0.97* | -0.16 |

# References

1. Marandel F, Charrier G, Lamy JB, Le Cam S, Lorance P, Trenkel VM: **Estimating effective population size using RADseq: Effects of SNP selection and sample size**. *Ecol Evol* 2020, **10**(4):1929–1937.

2. Pearman WS, Urban L, Alexander A: **Commonly used Hardy-Weinberg equilibrium filtering schemes impact population structure inferences using RADseq data**. *Mol Ecol Resour* 2022, **22**(7):2599–2613.

3. Andrews KR, Good JM, Miller MR, Luikart G, Hohenlohe PA: **Harnessing the power of RADseq for ecological and evolutionary genomics**. *Nat Rev Genet* 2016, **17**(2):81–92.

4. Bresadola L, Link V, Buerkle CA, Lexer C, Wegmann D: **Estimating and accounting for genotyping errors in RAD-seq experiments**. *Mol Ecol Resour* 2020, **20**(4):856–870.

5. Purcell S, Neale B, Todd-Brown K, Thomas L, Ferreira MA, Bender D, Maller J, Sklar P, De Bakker PI, Daly MJ: **PLINK: a tool set for whole-genome association and population-based linkage analyses**. *The American journal of human genetics* 2007, **81**(3):559–575.

6. Pearman WS, Urban L, Alexander A: **Commonly used Hardy–Weinberg equilibrium filtering schemes impact population structure inferences using RADseq data**. *Molecular Ecology Resources* 2022, **22**(7):2599–2613.

7. Wahlund S: **Zusammensetzung von population und korrelationserscheinung vom standpunkt der vererbungslehre aus betrachtet**. *Hereditas* 1928, **11**:65–106.
